# Single-cell analysis reveals immune remodeling of monocytes, NK cells, T cell exhaustion, and Galectin-9–associated depletion of gamma delta and mucosal-associated invariant T cells in Long COVID with ME/CFS

**DOI:** 10.3389/fimmu.2026.1745933

**Published:** 2026-02-25

**Authors:** Shima Shahbaz, Najmeh Bozorgmehr, Amirhossein Rahmati, Amal Abouda, Hussain Syed, Mohammed Osman, Shokrollah Elahi

**Affiliations:** 1 Mike Petryk School of Dentistry, Division of Foundational Sciences, University of Alberta, Edmonton, AB, Canada; 2 Department of Medicine, Division of Gastroenterology, University of Alberta, Edmonton, AB, Canada; 3 Department of Medicine, Division of Rheumatology, University of Alberta, Edmonton, AB, Canada; 4 Li Ka Shing Institute of Virology, University of Alberta, Edmonton, AB, Canada; 5 Women and Children Health Research Institute, University of Alberta, Edmonton, AB, Canada; 6 Cancer Research Institute of Northern Alberta, University of Alberta, Edmonton, AB, Canada; 7 Glycomics Institute of Alberta, University of Alberta, Edmonton, AB, Canada; 8 Alberta Transplant Institute, Faculty of Medicine and Dentistry, University of Alberta, Edmonton, AB, Canada

**Keywords:** olfactory receptors, platelets, Reelin, sex hormone receptors, TIM-3, Tregs

## Abstract

**Introduction:**

The cellular mechanisms underlying Long COVID (LC) associated with myalgic encephalomyelitis/chronic fatigue syndrome (ME/CFS) remain poorly understood.

**Methods:**

We performed single-cell RNA sequencing (scRNA-seq) on peripheral blood mononuclear cells collected 12 months after acute COVID-19 infection from female individuals with LC-ME/CFS and recovered (R) individuals. Comparative analysis was also performed using publicly available scRNA-seq datasets from idiopathic ME/CFS patients.

**Results:**

Based on transcriptional signatures, LC-ME/CFS patients exhibited a marked reduction in naïve CD4^+^ and CD8^+^ T cells, regulatory T cells, MAIT cells, and γδ T cells, accompanied by an expansion of effector T cells. NK cells displayed reduced frequency and altered activation-associated transcriptional factors, consistent with impaired cytotoxic potentials. B cells in LC patients exhibited gene expression profiles indicative of heightened activation, while plasma cells revealed a distinct transcriptional subset expressing NK-associated genes. Platelets and low-density neutrophils were expanded and exhibited enrichment of activated-related transcripts. Monocyte subsets demonstrated transcriptional skewing characterized by reduced expression of phagocytosis-associated genes and increased expression of pro-inflammatory cytokine-related genes/pathways. In contrast, idiopathic ME/CFS patients exhibited less pronounced immune alterations at the transcriptional level: while T cell activation was evident, there was no reduction in MAIT or NK cells, nor signs of T cell exhaustion. Notably, FOXP3 expression was upregulated, and B cells and platelets demonstrated dysregulated signatures in idiopathic ME/CFS. Mechanistically, we identify Galectin-9–TIM-3 interaction as a potential pathway driving γδ and MAIT cell depletion in LC.

**Conclusion:**

Our results reveal extensive peripheral immune remodeling in LC-ME/CFS, distinct from idiopathic ME/CFS, and support a model of chronic immune activation and dysregulation. Our findings offer a cellular framework for understanding LC pathogenesis and point to potential biomarkers and therapeutic targets for intervention.

## Introduction

Long COVID (LC), also referred to as post-acute sequelae of SARS-CoV-2 infection (PASC), represents a growing global public health concern characterized by lingering symptoms lasting for months and years beyond the acute phase of infection (1). Among the diverse clinical manifestations of LC, a subset of patients presents with the most debilitating symptoms closely resembling those observed in myalgic encephalomyelitis/chronic fatigue syndrome (ME/CFS), including profound fatigue, cognitive dysfunction, post-exertional malaise (PEM), and autonomic instability (2, 3). This overlap has prompted investigations into potential shared pathophysiological mechanisms between LC and ME/CFS, with an emphasis on immune, metabolic, and neurological dysregulation.

A wide range of immunological dysregulation and metabolomic alterations in LC patients, particularly those meeting diagnostic criteria for ME/CFS, have been reported. For example, metabolomic profiling has revealed distinct shifts suggestive of disrupted energy metabolism and mitochondrial dysfunction in LC patients (4). Concurrently, elevated plasma levels of multiple pro-inflammatory cytokines and chemokines reflect a state of chronic immune activation in LC patients, including those with ME/CFS (3, 5). Notably, the neurotrophic factors Artemin (ARTN) and Reelin, along with the immunomodulatory lectin Galectin-9 (Gal-9), were significantly increased in the plasma of LC patients with ME/CFS, highlighting their potential utility as diagnostic biomarkers (2, 3). Gal-9, an immunomodulatory lectin, exerts its effector functions through interactions with multiple receptors, including TIM-3 (6). The Gal-9–TIM-3 interaction has been associated with T cell apoptosis, T cell exhaustion, and impaired NK cell effector function (7–9). Inflammatory markers such as serum amyloid A (SAA) and C-reactive protein (CRP) were also elevated, supporting a systemic inflammatory milieu in these patients even 12 months after the initial acute disease (3).

Additionally, immunophenotyping analyses have revealed a skewed T cell landscape in LC. Specifically, a marked reduction in naive T cells, accompanied by an expansion of effector and terminal effector T cell subsets, indicating ongoing immune activation, which may promote T cell exhaustion in LC patients (3, 10). Furthermore, a depletion of key regulatory populations, including regulatory T cells (Tregs), natural killer (NK) cells, and mucosal-associated invariant T (MAIT) cells, alongside increased frequencies of neutrophils and monocytes, features consistent with immune dysregulation in LC patients (3, 11).

Additionally, LC patients exhibit expansion of CD71+ erythroid cells (CECs) and increased red cell distribution width (RDW), suggestive of dysregulated erythropoiesis, as reported in acute COVID-19 disease (12, 13). One potential underlying mechanisms associated with LC pathogenesis has been linked to viral persistence (14); however, this may occur in immunocompromised (15) but not in immunocompetent individuals (16). Instead, elevated plasma levels of biomarkers associated with impaired gut barrier integrity, including soluble CD14 (sCD14), intestinal-fatty acid-binding protein (I-FABP), and lipopolysaccharide-binding protein (LPS-BP) (2, 3), suggest microbial translocation as a possible driver of chronic immune activation in LC patients.

To further dissect the molecular underpinnings of LC with ME/CFS, we recently conducted bulk RNA sequencing (RNAseq) of whole blood samples from LC patients with ME/CFS at least 12 months post-infection, comparing them to fully recovered individuals. This analysis revealed a distinct transcriptional profile characterized by upregulation of genes involved in immune regulation and neuronal development, including *Fezf2, BRINP2, HOXC12, MEIS2, ZFHX3*, and *RELN*. Many of these genes have been implicated in neuroinflammation, cognitive function, and hematopoietic regulation, providing a mechanistic link to the clinical symptoms observed in LC (11).

Of particular interest, *RELN*, which encodes the Reelin protein, was significantly elevated in LC patients and may serve as a key biomarker of LC pathogenesis due to its dual roles in neural plasticity and immune modulation (17, 18). Although bulk RNAseq is valuable and informative, the heterogeneity of the immune and non-immune cells in the whole blood and the presence of exosomes and other tissue associated RNAs complicates to identify the source of altered genes. Therefore, single-cells RNAseq (scRNAseq) will enable an unbiased quantification of gene expression in different immune cell subsets.

In this study, we conducted scRNAseq on freshly isolated peripheral blood mononuclear cells (PBMCs) from LC patients with ME/CFS and recovered individuals (R) following SARS-CoV-2 infection. We show alterations in the frequency and functionality of different immune cell subsets. Collectively, our findings present global and dynamic immune response unique to each cell type in LC patients compared to the R group. We also reanalyzed publicly available scRNAseq from idiopathic ME/CFS patients (19) for comparison.

## Materials and methods

### Study design

A total of 20 human participants were enrolled for this study. Given that our LC cohort consists of over 75% females, we recruited ten female participants with LC/ME/CFS, with a median age of 48.25 ± 8.55 years. The control group included ten female individuals who had recovered (R) from acute COVID-19 disease without any persistent symptoms or complications, with a median age of 47.10 ± 10.3 years. All participants had their SARS-CoV-2 infection confirmed by PCR testing conducted at the University of Alberta Hospital (Edmonton, Canada). Both the LC and R cohorts were recruited approximately 12 months after the initial onset of infection. Freshly isolated PBMCs from five participants from each group were subjected to scRNAseq. In addition, PBMCs and plasma from these participants, together with samples from an additional five participants per group, were used for mechanistic studies. LC patients with ME/CFS were selected based on clinical evaluation, laboratory tests, and the administration of seven validated questionnaires consistent with the Canadian Consensus Criteria (CCC) for ME/CFS and WHO guidelines, as we have reported before (3, 11). Especially, we used the DePaul Symptom Questionnaire (DSQ) to identify patients meeting ME/CFS criteria, followed by assessments of fatigue severity using the FACIT Fatigue Scale (Version 4) and the Multidimensional Fatigue Inventory. A DSQ score of at least 5 out of 6 on key symptoms, such as PEM was required for ME/CFS diagnosis.

We excluded patients with confounding health conditions or those with the history of ICU admission at the time of acute SARS-CoV-2 infection or co-morbidities. Additionally, as detailed in our previously published work (3, 20), LC patients and R were carefully matched for age, sex, BMI, and time from infection, and individuals with underlying medical conditions were excluded. Based on this cohort design and the limited sample size, additional covariate regression using a DESeq2 design formula was not performed.

### Ethics statement

This study was approved by the Human Research Ethics Board (HREB) at the University of Alberta (Protocol# Pro00099502), and written informed consent was obtained from all participants prior to enrollment.

### Sample processing and cell culture

Freshly collected whole blood was processed within 30 min following venipuncture. Briefly, blood was centrifuged at 300 × g for 10 min to collect plasma, which was aliquoted into cryogenic tubes. The remaining blood was diluted 1:1 with sterile PBS and layered onto 15 mL Ficoll Paque gradients (GE Healthcare, Chicago, IL, USA) in a 50 mL conical tube, followed by centrifugation at 300 × g for 20 min at room temperature (RT) with no brake. The buffy coat was collected and washed twice in RPMI 1640 (Sigma‐Aldrich, Burlington, MA, USA) supplemented with 10% FBS (Sigma‐Aldrich), and 1% penicillin/streptomycin (Sigma‐Aldrich) at 300 × g at RT. Immediately after washing, PBMCs were either fixed for scRNAseq or subjected to flow cytometric analysis or functional assays according to our standard laboratory protocols (21, 22). In some experiments, PBMCs were cultured without or with human recombinant Gal-9 (rGal-9; 0.5 μg/ml, Gal Pharma) overnight for apoptotic assay.

### Flow cytometry analysis

Fluorophore-labeled antibodies with specificity to human cell antigens were purchased from BD Biosciences and Thermo Fisher Scientific. The following Abs were used in our study: anti-CD3 (SK7), anti-CD4 (RPA-T4), anti-CD8 (RPA-T8), anti-TIM-3 (7D3), anti-CD26 (M-A261), anti-CD161(HP-3G10), anti-TV α7.2 (3C10), anti-IL-18R α (H44), and anti TCRγδ (B1). To exclude dead cells, live/dead fixable Aqua staining (Thermo Fisher Scientific cat # L34965) was used. For defining the gating strategy and antibody specificity, appropriate FMO (fluorescence minus one) and isotype control antibodies were used per the supplier’s recommendation. Cells were stained in 96-well round-bottom plates at 4°C for 20 min using a live/dead stain, followed by 30 min incubation at 4 C with antibodies against other surface markers. Annexin V (BD Biosciences) was used to assess apoptosis in immune cells. To prevent variations, we used consistent flow cytometry panels with the same gating strategy for all study subjects, and a minimum of 100,000 events was acquired for each cell subset according to our protocols (23–25). Also, BD Biosciences CompBeads were used for control compensation. Following staining, cells were fixed and acquired on an LSRFortessa-SORP or Fortessa- X20 (BD Biosciences) and analyzed using the FlowJo software (version 10).

### ELISA assay

Frozen plasma samples at -20°C were thawed and centrifuged for 15 min at 1500g followed by dilution for Gal-9 concentration using the ELISA kit (R&D, DY 2045).

### RNAseq sample processing

PBMCs were fixed using the Fixation Kit (Parse Biosciences, Seattle, WA, USA) according to the manufacturer’s protocol. After fixation, the cell count was determined using the Countess 3 Automated Cell Counter (Thermo Fischer Scientific, Waltham, MA, USA). A total of approximately 322,000 cells were used from 10 donors, resulting in an average of approximately 32,200 cells per donor. These cells were subjected to the Evercode WT V3 single cell whole transcriptome kit (Parse Biosciences, Seattle, WA, USA) for library construction. The scRNA-seq libraries were sequenced using the Illumina NovaSeq X Plus platform, at a sequencing depth of approximately 35,000 reads per cell. Sequencing data was aligned and quantified against the GRCh38 human reference genome using TRAILMAKER pipeline v1.4.1. The resulting filtered count matrix was subsequently used for downstream analysis. The scRNA-seq data generated in this study are made publicly available under SRA accession number PRJNA1257454.

### Quality control and clustering

All quality control procedures were performed using Seurat v5.1.0 within the R statistical environment (v4.4.2). A Seurat object was created using the CreateSeuratObject function, including genes expressed in at least 3 cells and cells expressing a minimum of 200 genes. The object was then filtered using the subset function to remove potential doublets or multiplets (cells with > 4000 detected genes), as well as low-quality or dying cells with a high proportion of mitochondrial gene expression (> 5%). Individual datasets were merged using the merge function. To correct for batch effects across samples, dataset integration was performed using the Harmony package via the *RunHarmony* function (26) and the donor identity was retained. Data normalization and variance stabilization were carried out using the SCTransform function, specifying 3000 highly variable features. Dimensionality reduction was carried out using the RunPCA, FindNeighbours, and FindClusters functions in succession, retaining 25 principal components (PCs) as determined by inspection of the PCA elbow plot. Clustering was performed by varying the resolution parameter from 0 to 1 in increments of 0.1 using the FindClusters function. The optimal clustering resolution of 0.5 was selected based on the clustering hierarchy visualized with the Clustree package (27).

### Gene set comparisons

Differential gene expression analysis was performed by comparing the LC and R libraries. Upregulated and downregulated differentially expressed genes (DEGs) were identified using the FindAllMarkers function in Seurat, with parameters set to min.pct = 0.3 and logFC threshold = 0.3. A total of 227 DEGs with adjusted *p*-values< 0.05 were retained for downstream analysis. Heatmaps were generated using the DoHeatmap function to visualize DEG expression patterns. Additional visualization methods included density plots (DensityPlot), feature plots (FeaturePlot), violin plots (VlnPlot), ridge plots (RidgePlot), and dot plots (DotPlot), each used to display gene expression distributions and marker profiles across clusters.

Common exhaustion genes (CTLA4, TOX, EOMES, TIGIT, PDCD1, LAG3, HAVCR2, CD160, ENTPD1, and BATF) (28, 29), MAIT activation genes (KLRB1, IL7R, STAT1, STAT3, STAT4, CD69, RORA, JUN, JUNB, and SELL) (30), and NK activation genes (NKG7, KLRD1, NCAM1, PRF1, IFNG, IL2RB, GZMB, GNLY, NCR1, KLRF1, BCL2, FCGR3A, CD69, GZMA, LAMP1, KLRB1, CASP8, and RIPK1) (31, 32) were used to calculate T cell exhaustion, MAIT, and NK activation scores, respectively.

### Gene Set Enrichment Analysis (GSEA) in Single-cell clusters

Single-cell gene set enrichment analysis was performed to identify differentially enriched pathways between LC and R groups within each cell cluster. For each qualifying cluster, pseudo bulk expression profiles were generated by aggregating raw gene counts across biological replicates within each condition. Differential expression between LC and R was assessed using edgeR with a quasi-likelihood framework. Genes were ranked by a signed significance metric, the sign of the log fold change multiplied by the negative log10 of the raw p-value. This approach prioritizes genes with large directional changes and high statistical confidence. GSEA was carried out using the fgsea package in R. Pathways were sourced from the MSigDB Hallmark collection and Reactome pathways. Only pathways with an adjusted p-value (padj)< 0.25 were retained. Normalized Enrichment Scores (NES), nominal p-values, adjusted p-values, and leading-edge genes were computed and reported for each cluster. Positive NES values indicate pathway upregulation in LC, while negative NES values indicate downregulation. For visualization at the compartment level, immune cell clusters were grouped into adaptive and innate compartments based on canonical lineage identity. NES for selected Hallmark pathways were summarized within each compartment using significance-weighted averaging across constituent cell types. Positive NES values indicate pathways enriched in LC relative to the R controls.

### Statistical analysis and bioinformatics

Data distribution was evaluated using the Shapiro–Wilk test. For comparisons involving non-normally distributed data, statistical significance was assessed using the Mann–Whitney U test or the Wilcoxon matched-pairs signed-rank test, as appropriate. Results are presented as mean ± SEM, and P values< 0.05 were considered statistically significant. In violin plots, the central line denotes the median, while the lower and upper boundaries correspond to the first and third quartiles, respectively. No randomization procedures were applied, and no data points were excluded from the analyses.

Differential gene expression analysis was conducted on count-based data using the DESeq2 R package (R version 4.2.0). Genes were classified as differentially expressed if they met the criteria of log_2_ fold change greater than +1 or less than −1, together with an adjusted P value (Padj)< 0.05. Bubble plots were generated using custom Python scripts that integrate log_2_ fold change and adjusted P values, as previously described (11, 33).

## Results

### scRNA-seq profiling of PBMCs from LC and R individuals

**Figure 1:**
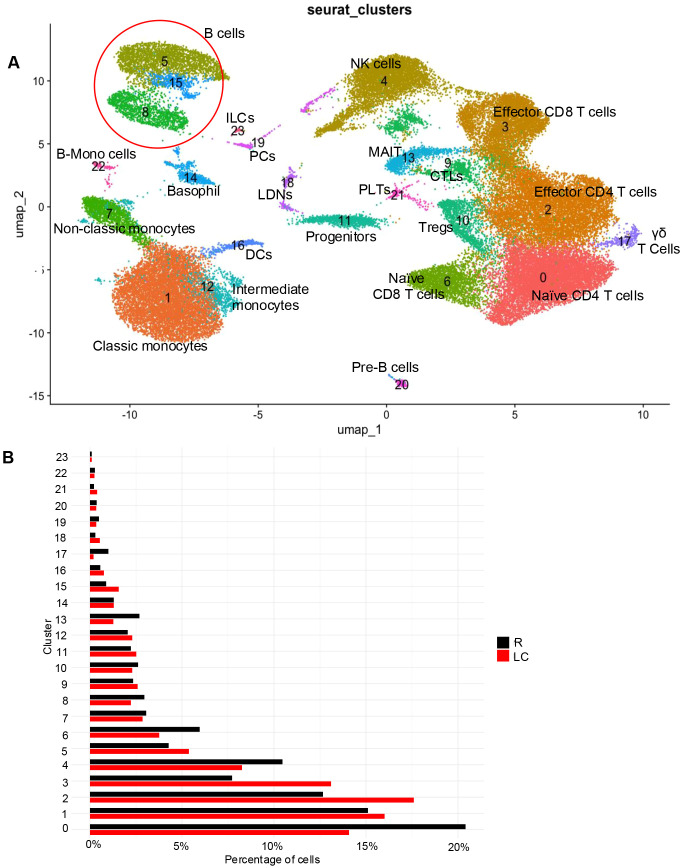
Characterization of immune cell clusters in PBMCs from R and LC individuals. **(A)** UMAP plot of 50,856 PBMCs, displaying major immune cell clusters, with each dot representing a single cell and colors indicating distinct cell types. **(B)** Bar plot showing the proportion of global immune cell clusters in PBMCs from R and LC individuals.

Freshly isolated PBMCs from ten individuals, five LC patients with ME/CFS and five recovered individuals (R) following acute SARS-CoV-2 infection without any LC symptoms, were processed for scRNAseq. LC patients with ME/CFS were diagnosed according to the criteria as outlined in the Canadian Consensus Criteria (CCC) for ME/CFS and WHO (34, 35). A total of 50,856 cells passed quality control and were retained for downstream analysis. We clustered cells using Seurat (33) based on their transcriptomic profiles and visualized them in two-dimensional space. This analysis identified 24 distinct cell clusters based on the expression of canonical marker genes (**Figure 1A**). Among T cells, CD4 T cells (clusters 0 and 2) were characterized by CD3E and CD4 expression, while CD8 T cells (clusters 3 and 6) expressed *CD3E*, *CD8A*, and *CD8B* (**Supplementary Figure 1A**). Tregs (cluster 10) were defined by *FOXP3* (**Supplementary Figure 1B**), whereas cytotoxic T lymphocytes (CTLs; cluster 9) exhibited *TBX21* expression (**Supplementary Figure 1C**). MAIT cells (cluster 13) were annotated based on *KLRB1*, *IL18RAP*, *RORC*, and *ZBTB16* genes (**Supplementary Figures 1D, E**), while gamma delta (γδ) T cells (cluster 17) were identified by *TRDC*, *TRGC1*, and *TRGC2* genes (**Supplementary Figure 1F**). Additionally, *NKG7*, *GNLY*, and *NCAM1* genes were used to identify NK cells (cluster 4) (**Supplementary Figure 1G**) and *CD79A* gene to recognize B cells (clusters 5, 8, 15) (**Supplementary Figure 1H**).

Monocytes were subdivided into CD14+ monocytes (clusters 1, 12), characterized by the expression of *S100A8* and *CD14* as well as non-classical monocytes (cluster 7), distinguished by *CDKN1C* gene (**Supplementary Figure 1I**). Intriguingly, we identified a B-monocyte (B-Mono) cell (cluster 22) exhibited markers of both B cells and monocytes. Other identified blood cell types included platelets (PLTs; cluster 21), defined by *TUBB1* expression (**Supplementary Figure 1J**), dendritic cells (DCs; cluster 16), characterized by *ITGAX* and *CLEC10A* (**Supplementary Figure 2A**), and basophils (Baso; cluster 14), identified by *HDC* expression (**Supplementary Figure 2B**). Plasma cells (PCs; cluster 19) were marked by *JCHAIN* and *IGHA1* (**Supplementary Figure 2C**), while low-density neutrophils (LDNs; cluster 18) were defined by *LTF* (**Supplementary Figure 2D**). Furthermore, hematopoietic stem and progenitor cells (HSPCs; cluster 11) and innate lymphoid cells (ILCs; cluster 23) were classified as lineage-negative populations (36) (**Supplementary Figure 2E**). HSPCs were identified by *HNRNPA1* and *TMSB4X*, whereas ILCs were marked by *KIT* expression (**Supplementary Figure 2E**). We additionally validated our cell type annotations through computational reference-based classification using SingleR (**Supplementary Figure 2F**). These findings provide a comprehensive overview of the major immune cell subsets identified through clustering analysis of PBMCs.

### A relative decrease in MAIT, γδ, naive CD4, and CD8 T cells in LC patients

We next compared the frequency of each immune cell cluster between R and LC patients by calculating its percentage relative to the total immune cell population within each group, as determined by scRNAseq analysis (**Figure 1B**, **Supplementary Figure 3A**). These analyses revealed the enrichment of clusters effector 2 (effector CD4 T cells), 3 (effector CD8 T cells), 5 (a subcluster of B cells), 15 (a subcluster of B cells), 16 (DCs), 18 (LDNs), and 21 (PLTs) and depletion of clusters 0 (naïve CD4 T cells), 6 (naïve CD8 T cells),13 (MAIT cells), and 17 (γδ T cells) in LC patients, while other subsets show negligible changes (**Figure 1B**). When we compared the clusters based on cell numbers, we observed a similar pattern (**Supplementary Figure 3B**). These observations suggest differential effects of LC on the frequency of various immune cell subsets.

### Differential transcriptional effects of LC across immune cell subsets

**Figure 2:**
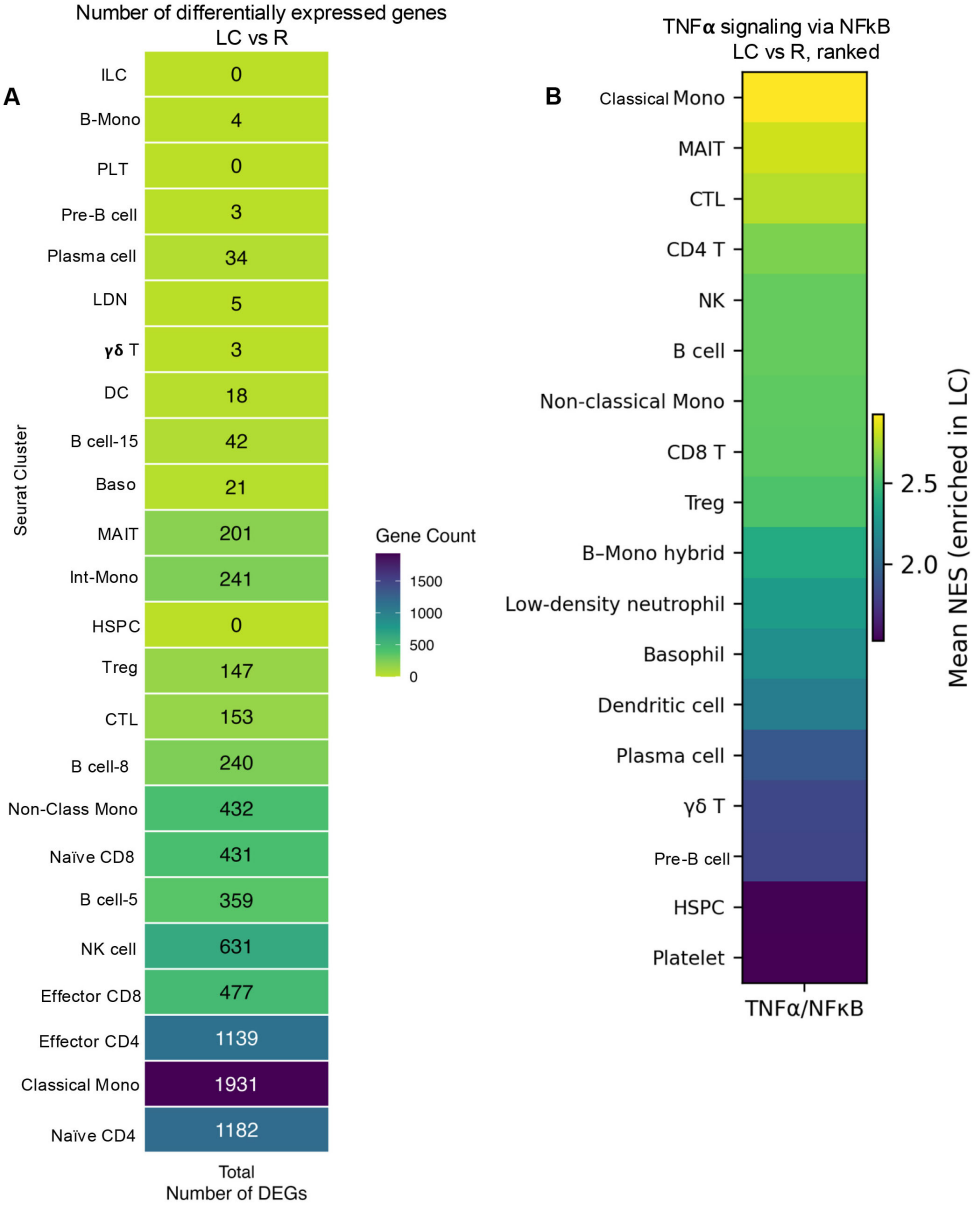
Number of differentially expressed genes and TNF-α signaling via NFkB in LC vs R individuals. **(A)** The number of differentially expressed genes (log2 FC> 0.25, FDR< 0.05) per cluster in the LC vs. the R group. **(B)** Heatmap showing mean normalized enrichment scores (NES) for the Hallmark TNFα signaling via NFκB pathway across annotated immune cell types, ranked from highest to lowest enrichment. Positive NES values indicate pathway enrichment in LC patients relative to R individuals. Innate lymphoid cells (ILCs), B-monocytes (B-Mono), Platelets (PLTs), Low density neutrophils (LDNs), Dendritic cells (DCs), B cells; cluster 15 (B cells-15), Basophil (Baso), intermediate-monocytes (Int-Mono), Hematopoietic stem and progenitor cells (HSPCs), Cytotoxic T lymphocytes (CTLs), B cell cluster 5 (B cell-5), and B cell cluster 8 (B cell-8).

To further assess the biological impact of LC with ME/CFS across immune cell populations, we quantified the number of significantly differentially expressed genes per cell cluster. This analysis revealed that classical monocytes (cluster 1) were the most transcriptionally affected subset, followed by CD4 T cell subsets (clusters 0 and 2), NK cells (cluster 4), CD8 T cell subsets (clusters 3 and 6), and non-classical monocytes (**Figure 2A**). Collectively, these findings indicate that LC exerts differential transcriptional effects across distinct immune cell types.

### Cell-type–specific pathway alterations in LC patients

We performed GSEA on cluster-specific differentially expressed genes derived from scRNAseq data. Across immune compartments, LC patients exhibited distinct yet partially overlapping transcriptional pathway alterations, with enrichment of TNF-α signaling via NF-κB, interferon-associated, inflammatory, and metabolic pathways, indicating widespread immune remodeling in LC (**Supplementary Table 1**, **Supplementary Figure 4A**).

CD4^+^ T cells (clusters 0 and 2) showed significant enrichment of TNFα signaling via NFκB and IFN-γ response pathways in LC patients compared with R individuals. In addition, pathways related to RNA processing and mRNA splicing were upregulated, consistent with increased transcriptional activity (**Supplementary Table 1**, **Supplementary Figure 4A**). CD8^+^ T cells (clusters 3 and 6) similarly demonstrated enrichment of interferon-associated and inflammatory pathways, accompanied by modulation of metabolic programs, including oxidative phosphorylation in select subsets. These transcriptional changes reflected altered activation states within CD8^+^ T cells in LC (**Supplementary Table 1**, **Supplementary Figure 4A**). Tregs (cluster 10) exhibited enrichment of interferon-associated and activation-related pathways in LC patients, suggesting transcriptional remodeling and instability within immunoregulatory compartments. CTLs (cluster 9) also showed enrichment of IFN-γ–responsive and inflammatory pathways, indicating persistent cytokine-associated transcriptional activity (**Supplementary Table 1**, **Supplementary Figure 4A**). Similarly, MAIT cells (cluster 13) and γδ T cells (cluster 17) displayed prominent enrichment of interferon-related pathways in LC patients (**Supplementary Table 1**, **Supplementary Figure 4A**). NK cells (cluster 4) showed enrichment of interferon-responsive and inflammatory pathways in LC patients. B-cell subsets (clusters 5, 8, and 15) demonstrated enrichment of inflammatory and complement-associated pathways in LC patients, together with modulation of metabolic programs in selected clusters (**Supplementary Table 1**, **Supplementary Figure 4A**). In contrast, plasma cells (cluster 19) exhibited downregulation of oxidative phosphorylation and metabolic pathways, indicating altered metabolic activity within antibody-secreting cell populations in LC (**Supplementary Table 1**, **Supplementary Figure 4A**). Notably, CD14^+^ monocytes (clusters 1 and 12) displayed some of the most extensive transcriptional alterations in LC patients. These cells exhibited strong enrichment of TNFα/NFκB signaling, type I and type II interferon responses, and complement activation pathways, along with increased transcriptional and RNA-processing programs (**Supplementary Table 1**, **Supplementary Figure 4A**). Non-classical monocytes (cluster 7) also demonstrated enrichment of activation- and inflammation-associated pathways, although to a lesser extent than classical monocytes and B–monocyte hybrid population (cluster 22) showed mixed innate and adaptive transcriptional signatures, consistent with altered immune-state plasticity (**Supplementary Table 1, Supplementary Figure 4A**). LDNs (cluster 18) exhibited strong enrichment of both type I and type II interferon pathways, together with inflammatory related gene sets and DCs (cluster 16) demonstrated altered activation- and antigen-processing–associated pathways, suggesting transcriptional remodeling of antigen-presenting functions (**Supplementary Table 1**, **Supplementary Figure 4A**). Finaly, Basophils (cluster 14), despite low abundance, also displayed activation-associated transcriptional signatures (**Supplementary Table 1**, **Supplementary Figure 4A**).

Notably, heatmap visualization of TNFα signaling via NFκB as the dominant enriched pathway across immune cell clusters revealed the highest pathway enrichment in classical monocytes, MAIT cells, CTLs, CD4 T cells, and NK cells followed by intermediate enrichment in multiple lymphoid subsets, while platelets exhibited minimal pathway enrichment (**Figure 2B**).

To provide a higher-level view of pathway alterations across immune compartments, Hallmark pathway enrichment scores were summarized at the compartment level by grouping immune cell subsets into adaptive and innate populations. Radar plot visualization revealed broadly shared enrichment of inflammatory and interferon-associated pathways across both compartments in LC patients with ME/CFS (**Supplementary Figure 4B**). Specifically, TNFα signaling via NFκB, interferon-γ response, and inflammatory response pathways were enriched in both adaptive and innate immune cells. In contrast, type I interferon (IFN-α) signaling showed relatively stronger enrichment within innate immune compartments. IL-6–JAK–STAT3 signaling and oxidative phosphorylation pathways displayed more moderate and heterogeneous enrichment patterns across compartments (**Supplementary Figure 4B**). Together, this analysis highlights coordinated yet non-identical transcriptional pathway remodeling between adaptive and innate immune systems in LC.

### The unique source of selected genes associated with neuronal function

**Figure 3:**
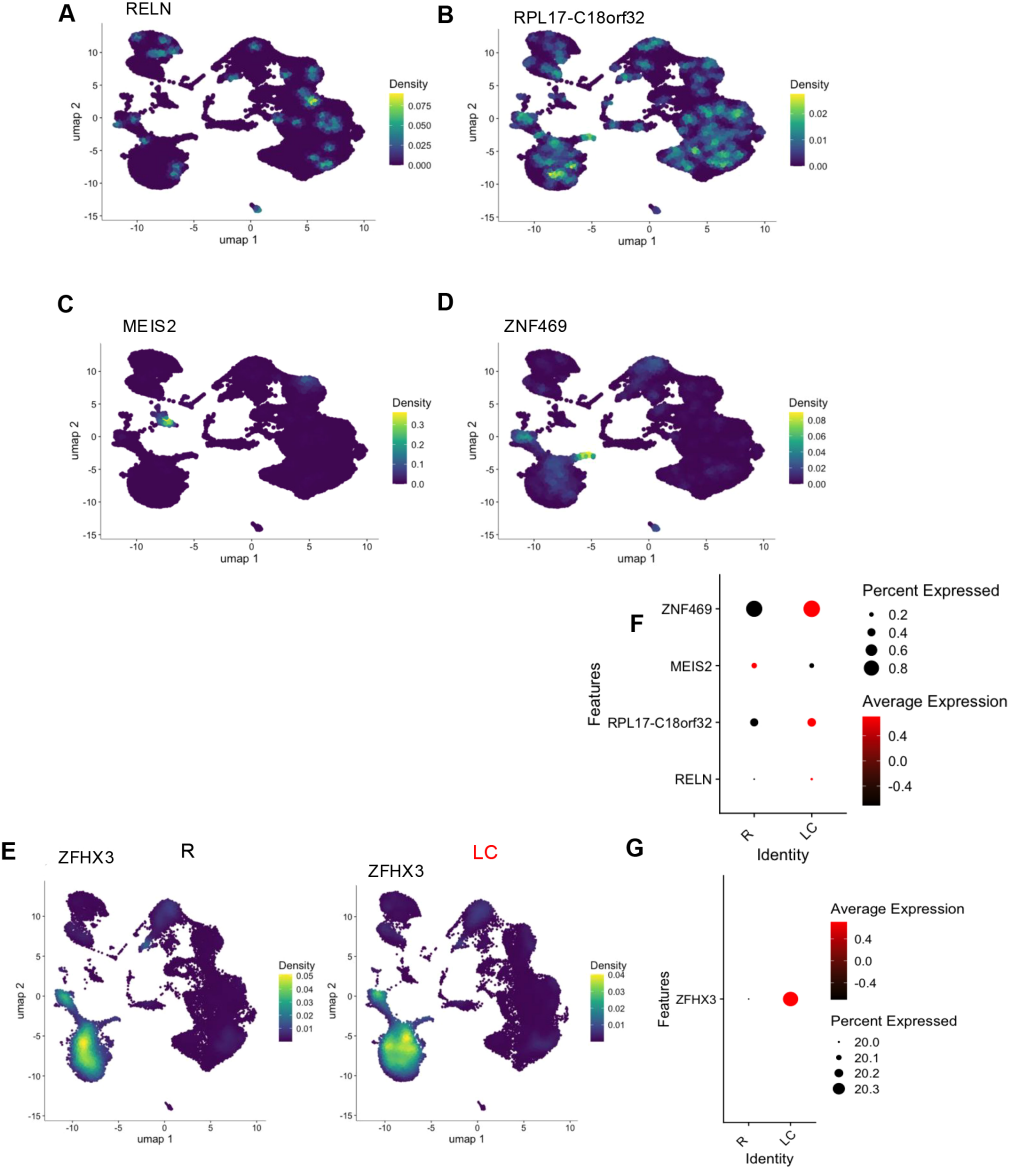
Expression pattern of selected genes associated with neuronal function across different immune subsets in LC and R individuals. Density plots depicting the distribution of **(A)** RELN, **(B)** RPL17-C18orf32, **(C)** MEIS2, **(D)** ZNF469, and **(E)** ZFHX3 gene expression across different clusters. **(F, G)** Bubble plots illustrating the expression patterns of the above genes in R and LC individuals. **(A–D)** represent analyses of pooled cells, while **(E)** is shown separately because of its substantial and distinct expression pattern.

In our previous study when bulk RNAseq on the whole blood was performed (11), we described the top 10 upregulated and top 10 downregulated genes in LC patients compared to the R group. Given that whole blood contains RNA from diverse tissue sources, we questioned whether these genes were detectable in PBMCs of LC patients. Among the upregulated genes, *RELN*, *RPL17-C18orf32*, *MEIS2*, *ZNF469*, and *ZFHX3* were detected in our scRNAseq dataset (**Figures 3A–G**). Notably, we found that *RELN* and *RPL17-C18orf32* were broadly expressed across various immune cell types (**Figure 3A, B**). While *MEIS2* expression was primarily observed in basophils (**Figure 3C**), *ZNF469* was predominantly expressed in DCs and non-classic monocytes (**Figure 3D**), and ZFHX3 was highly expressed in both types of monocytes (**Figures 3E, G**). Consistent with our bulk RNAseq findings, while *RELN*, *RPL17-C18orf32*, *ZFHX3*, and *ZNF469* were generally upregulated, MEIS2 expression was paradoxically downregulated in LC patients (**Figure 3F**).

**Figure 4:**
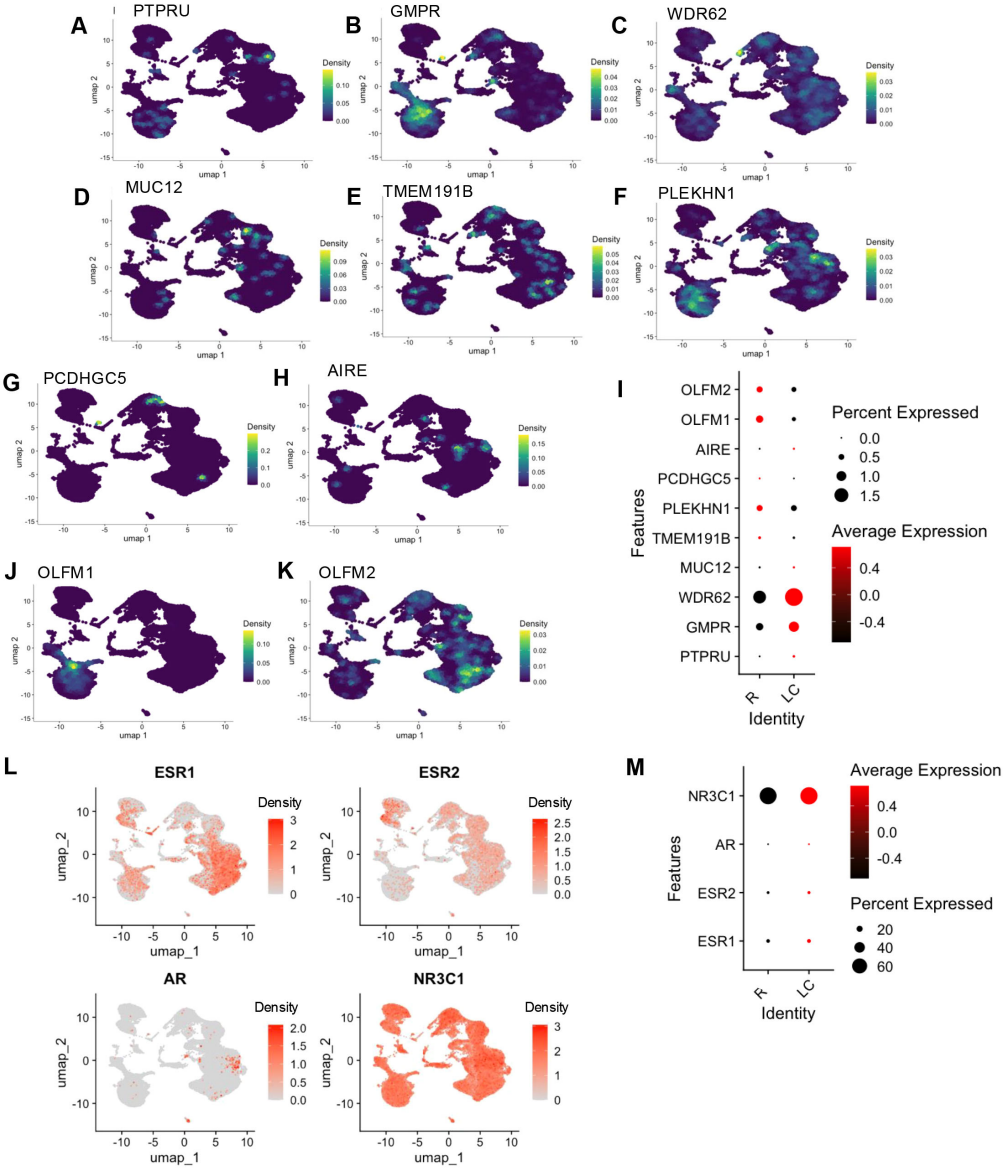
Expression profiles of neuronal, hormonal, and development-associated genes across PBMC clusters in R and LC individuals. Density plots showing the distribution of **(A)***PTPRU*, **(B)***GMPR*, **(C)***WDR62*, **(D)***MUC12*, **(E)***TMEM191B*, **(F)***PLEKHN1*, **(G)***PCDHGC5*, and **(H)***AIRE* across different immune clusters. **(I)** Bubble plot illustrating the expression patterns of these genes plus *OLFM1* and *OLFM2* in R and LC individuals. Density plots showing the distribution of **(J)***OLFM1* and **(K)***OLFM2* across different immune clusters. **(L)** Feature plots showing the expression of hormone-related receptor genes across different PBMC clusters; estrogen receptor 1 (ESR1) top left, ESR2 top right, androgen receptor (AR) bottom left, and the glucocorticoid receptor (NR3C1) bottom right. **(M)** Bubble plot showing the differential expression of hormone-related genes between R and LC individuals. UMAP distributions reflect pooled cells.

Next, we examined the expression of the top 10 downregulated genes in bulk RNAseq in scRNAseq dataset. Among these, *PTPRU*, *GMPR*, *WDR62*, *MUC12*, *TMEM191B*, *PLEKHN1*, *PCDHGC5*, and *AIRE* were detected and found to be broadly distributed across all cell clusters (**Figures 4A–H**). We found the downregulation of TMEM*191B*, *PLEKHN1*, and *PCDHGC5* in LC patients, which is consistent with our bulk RNAseq data. In contrast, the expression of *PTPRU*, *GMPR*, *WDR62*, *MUC12*, and *AIRE* were paradoxically upregulated in LC individuals (**Figure 4I**).

These observations highlight differential sources of specific genes in immune and non-immune cells.

### Downregulation of olfactory but upregulation of estrogen and androgen receptor genes in LC

Our whole blood RNAseq analysis revealed the upregulation of 25 olfactory receptor (OR) genes and downregulation of 2 olfactory-related genes (*OLFM1* and *OLFM2*) in LC patients (11). Intriguingly, none of these 25 OR genes were detected in our scRNAseq dataset. However, consistent with our bulk dataset, we observed downregulation of *OLFM1* and *OLFM2* in LC patients (**Figures 4I–K**). Interestingly, *OLFM1* was primarily expressed in monocytes, as well as DCs, while *OLFM2* showed diffuse distribution across PBMCs (**Figures 4J, K**). These findings indicate non-immune cell sources for detected 25 ORs in bulk RNAseq analysis (11).

**Figure 5:**
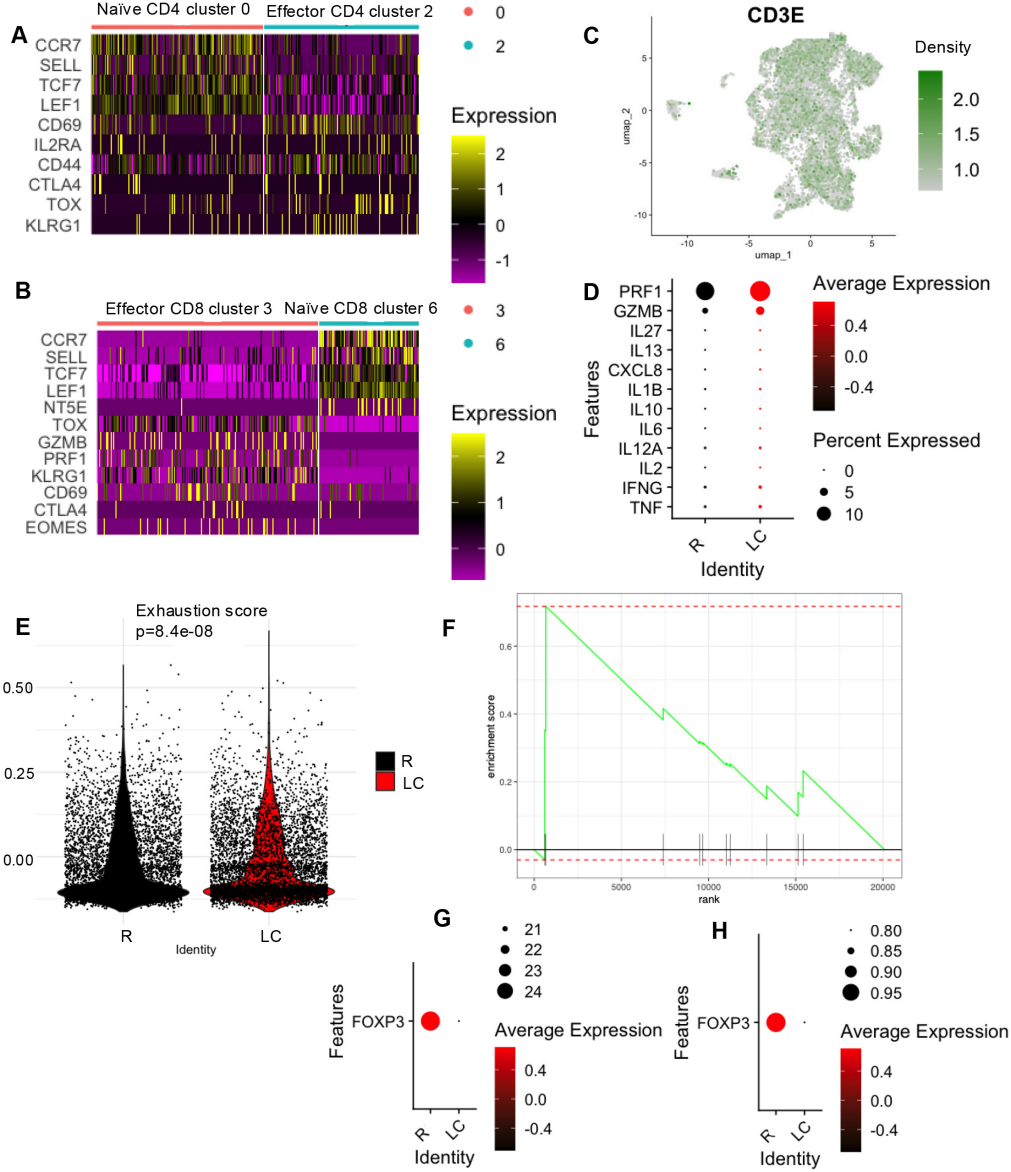
Comparative analysis of T cells and γδ T cell subsets in LC and R cohorts. **(A)** Heatmap illustrating gene expression profiles distinguishing naïve (cluster 0) from effector CD4 T cells (cluster 2). **(B)** Heatmap illustrating gene expression profiles distinguishing naïve (cluster 6) from effector CD8+ T cells (cluster 3). **(C)** Feature plot visualizing the spatial distribution of *CD3E*-positive cells (expression > 0.5). **(D)** Bubble plot depicting differentially expressed genes associated with T cell activation and cytotoxic mechanisms in LC compared to R individuals. **(E)** Distribution of T cell exhaustion module scores comparing LC and R individuals. **(F)** GSEA analysis shows exhaustion signature score among total CD8 T cells (clusters 3, 6 and 9) in LC patients. The green line is the running enrichment score for our exhaustion gene set (CTLA4, TOX, EOMES, TIGIT, PDCD1, LAG3, HAVCR2, CD160, ENTPD1, BATF) and the little black ticks on the x axis show where each of those exhaustion genes landed in the ranking. The very first genes (rank 1, 2, etc.) are the ones most strongly upregulated in LC with the highest confidence. Genes at the far right on the x axis (high rank numbers) are downregulated. Bubble plots showing *FOXP3* levels in **(G)** total PBMCs and **(H)** Tregs clusters, comparing LC and R individuals.

Additionally, our bulk RNAseq data had revealed the upregulation of hormone-related receptors, including estrogen receptors 1 and 2 (ESR1, ESR2), androgen receptor (AR), and the glucocorticoid receptor (NR3C1), in female LC patients compared to R females (11). Our scRNAseq analysis confirmed the same pattern in PBMCs of LC patients (**Figure 4L**). Notably, NR3C1, AR, and ESR2 showed the highest expression in the pre-B cell population (cluster 20), while ESR1 was highly expressed in plasma cells (cluster 19) (S **Figure 5A**). The expression levels of these genes were significantly upregulated in LC patients compared to the R group (**Figure 4M**). These findings highlight distinct transcriptional signatures in PBMCs of LC patients, including dysregulation of immune-related genes, olfactory signaling components, and hormone receptor pathways, suggesting complex immune and hormonal involvement in the pathophysiology of LC.

### LC is characterized by a reduction in naïve T cells and Tregs but an increase in effector T cells

We then compared changes in T cell populations between R and LC patients. As shown in **Figure 1A**, CD4^+^ T cells were represented by clusters 0 and 2, while CD8^+^ T cells corresponded to clusters 3 and 6. Closer examination revealed that cluster 0 expressed naïve CD4^+^ T cell marker genes such as *CCR7* and *SELL*, whereas cluster 2 expressed genes typically associated with effector CD4^+^ T cells, including *CD69*, *KLRG1*, and *TOX* (**Figure 5A**). Similarly, within the CD8^+^ T cell population, cluster 6 exhibited markers of naïve CD8^+^ T cells, while cluster 3 was enriched for genes linked to effector CD8^+^ T cells (**Figure 5B**).

Notably, clusters 2 and 3, representing activated CD4^+^ and CD8^+^ T cells, respectively, were expanded in LC patients, whereas clusters 0 and 6—representing naïve T cells—were depleted (**Figure 1B**, **Supplementary Figure 3B**). These findings may reflect a transition from naïve to effector states, potentially driven by increased immune activation in LC patients, as we previously reported by flow cytometry analysis (3).

Next, we subsetted cells with a CD3E expression level greater than 0.5 (**Figure 5C**) and compared the expression of cytokine genes typically secreted by T cells, as well as the genes encoding perforin (*PRF1*) and granzyme B (*GZMB*), two key cytotoxic molecules, between R and LC patients. In this subset, we observed higher expression of T cell-associated cytokine genes including *TNF*, *IFNG*, *IL2*, *IL12A*, *IL6*, *IL10*, *IL1B*, *CXCL8 (IL-8)*, *IL13*, *IL27, PRF1*, and *GZMB* compared to the R group (**Figure 5D**). Given that persistent immune activation may contribute to T cell exhaustion (37, 38), we observed a significantly higher exhaustion score in LC patients (**Figure 5E**), consistent with our previous findings (3). Additional GSEA analysis revealed significant enrichment of a CD8 T cell exhaustion-associated gene signature in clusters 3, 6, and 9 in LC compared with R controls, with exhaustion-related genes preferentially ranked among transcripts upregulated in LC (**Figure 5F**). Among the most highly ranked genes were CTLA4, ENTPD1, and TOX, respectively.

While our previous bulk RNAseq analysis revealed a reduction in Tregs subset in LC patients (11), the proportion of Tregs (cluster 10) appeared unchanged between LC and R groups in the current dataset (**Figure 1B**, **Supplementary Figure 3B**). However, further analysis revealed a substantial reduction in the expression of *FOXP3* gene—both across the entire PBMC population and specifically within the Treg cluster in LC patients (**Figures 5G, H**). These findings support our previous observations, highlighting increased T cell exhaustion and possibly impaired Treg function in LC patients.

Finally, to gain deeper insight into the biological impact of LC on different T cell subsets, we quantified the number of DEGs within each cluster. This analysis revealed that cluster 3 (effector CD8 T cells) exhibited the highest number of DE genes, followed by cluster 0 (naïve CD4 T cells) and naïve CD8 T cells (**Supplementary Figure 5B**). In contrast, LC was associated with a smaller number of transcriptional changes in cluster 9 (CTLs) and cluster 10 (Tregs) (**Supplementary Figure 5B**). These results demonstrate that effector CD8 T cells are disproportionately affected by LC at the transcriptional level.

### MAIT cells and γδ T cell are depleted in LC patients

**Figure 6:**
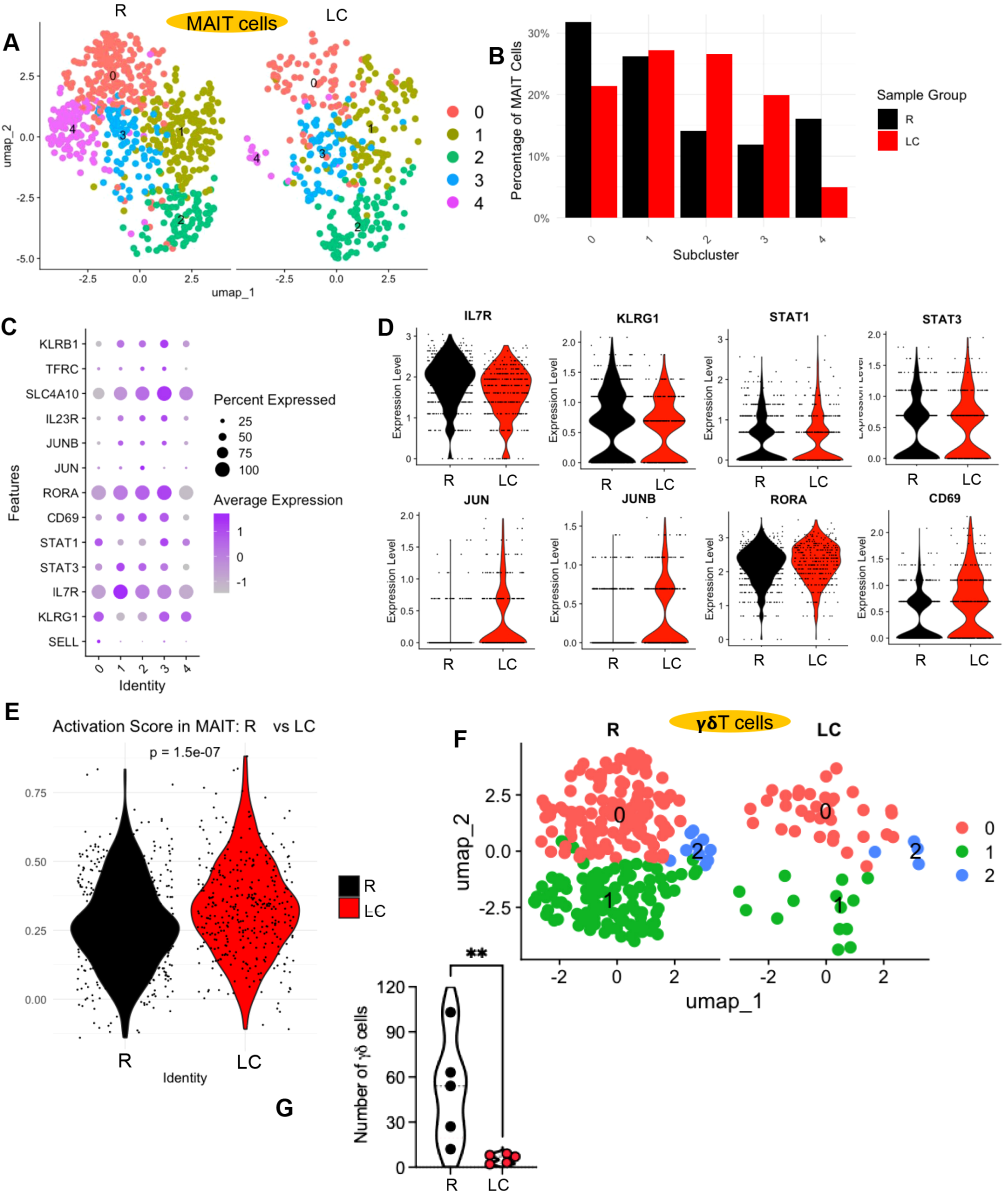
MAIT and γδ T cell subset characterization and activation profiles in LC versus R individuals. **(A)** UMAP projection showing different subsets of MAIT cells. **(B)** Bar plots showing the percentage distribution of MAIT cell subsets in LC and R individuals. **(C)** Bubble plot and **(D)** violin plots depicting differentially expressed genes associated with MAIT cell activation in LC compared to R individuals. **(E)** Distribution of MAIT cell activation module scores comparing LC and R individuals. **(F)** UMAP projection revealing distinct γδ T cell subpopulations in LC versus R individuals. **(G)** Cumulative data showing the number of detected γδ T cell in PBMCs in R and LC patients. P value was evaluated using the Mann–Whitney U test, **P< 0.01.

We then evaluated unconventional T cell populations, including MAIT and γδ T cells in patients with LC. We observed a reduction in MAIT cells, represented by cluster 13, in LC patients (**Figure 1B**, **Supplementary Figure 3B**). Further subsetting of this cluster revealed five MAIT cell subpopulations (**Figure 6A**). Interestingly, while the proportions of subclusters 0 and 4 as most dominant subsets were decreased, there was an increase in the proportion of subclusters 2 and 3 in LC patients (**Figure 6B**). Gene expression analysis revealed that subclusters 2 and 3 were enriched for genes associated with activated MAIT cells, such as *STAT1*, *STAT3*, *CD69*, *JUN*, *JUNB*, *RORA*, *IL23R*, *SLC4A10*, *TFRC*, and *KLRB1*, and exhibited lower expression of genes typically associated with a naïve MAIT phenotype, including *SELL*, *KLRG1*, and *IL7R* (**Figure 6C**). We also observed downregulation of *IL7R* and *KLRG1* and upregulation of *STAT1*, *STAT3*, *JUN*, *JUNB*, *RORA*, and *CD69* in the overall MAIT cell cluster in LC patients (**Figure 6D**). Consistently, MAIT cells also exhibited a higher activation score in LC patients (**Figure 6E**). These results suggest that the overall decrease in MAIT cells in LC patients is largely driven by a loss of naïve MAIT cells, while the proportion of activated MAIT cells relatively increased.

We also observed a reduction in γδ T cells in LC patients. Specifically, cluster 17, representing γδ T cells, was reduced in LC patients (**Figure 1B**, **Supplementary Figure 3B**). Further sub-setting of this cluster revealed three distinct γδ T cell subpopulations, all of which were depleted in LC patients (**Figures 6F, G**) with differential pattern of gene expression profile in these subsets (**Supplementary Figure 6A**). These findings suggest that LC may be associated with a reduction in multiple γδ T cell subsets.

### LC is associated with a reduction in NK cell frequency and function

**Figure 7:**
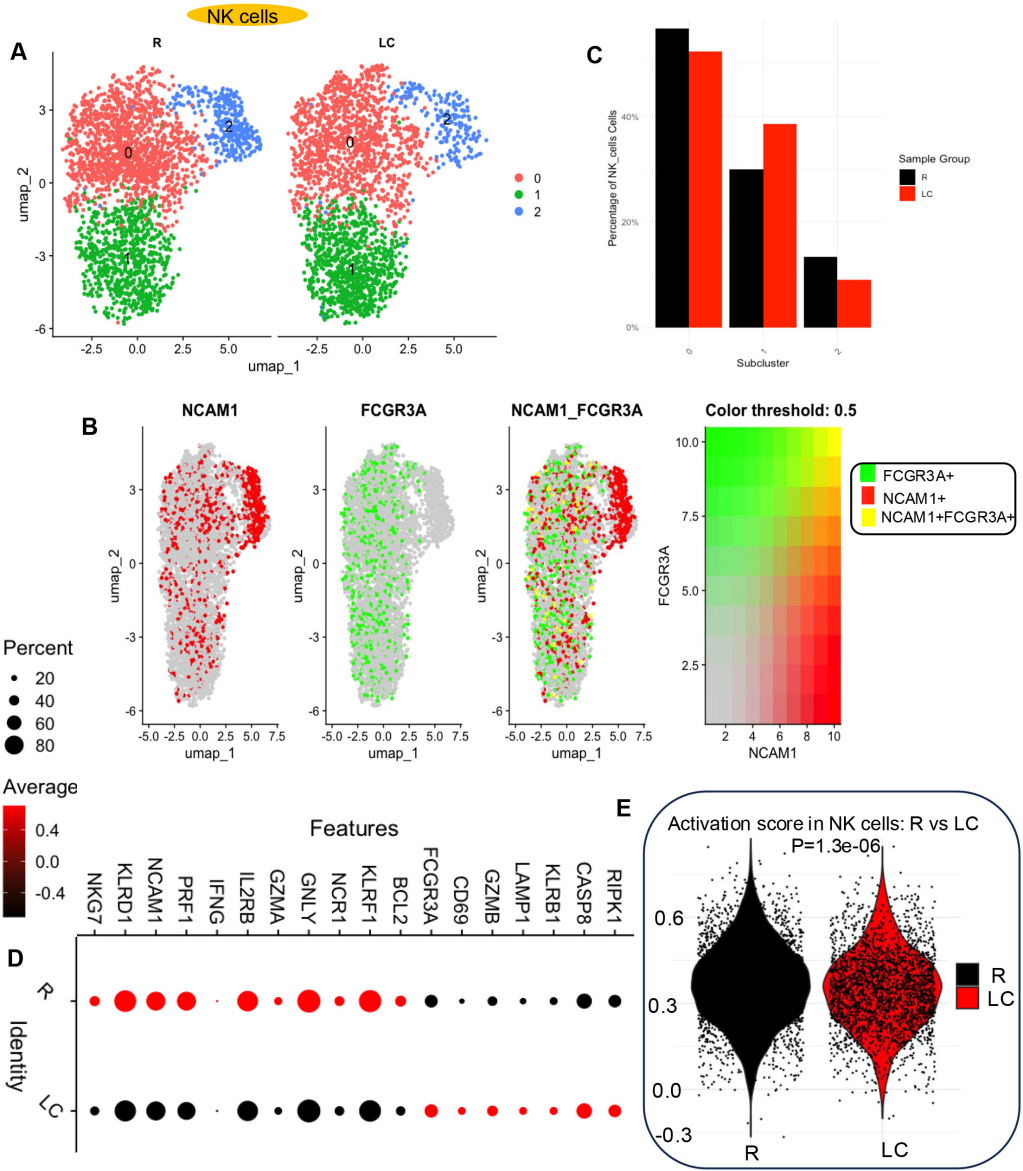
Comparative analysis of NK frequency and function in LC versus R individuals. **(A)** UMAP projection showing different subsets of NK cells. **(B)** Feature plots showing the co-expression of *NCAM1* and *FCGR3A* in NK cell clusters; the left panel shows NK cells expressing *NCAM1* (CD56), the middle panel shows NK cells expressing *FCGR3A* (CD16), and the right panel depicts cells co-expressing *NCAM1* and *FCGR3A* across NK subclusters. The color-threshold panel illustrates *FCGR3A* expression on the left and *NCAM1* expression on the right, highlighting a small subset of NK cells that co-express both markers. **(C)** Bar plots showing the percentage distribution of NK cell subsets in LC and R individuals. **(D)** Bubble plot depicting differentially expressed genes associated with NK cell activation in LC compared to R individuals. **(E)** Distribution of NK cell activation module scores in LC and R individuals.

Evaluation of the NK cell population (cluster 4) (**Figure 1A**) revealed a reduced frequency in LC patients, consistent with our previous bulk RNAseq data (11) (**Figure 1B**, **Supplementary Figure 3B**). Sub-clustering of the NK cell population within cluster 4 revealed three distinct NK cell subsets (**Figure 7A**). Examination of CD56 (*NCAM1*) and CD16 (*FCGR3A*) gene expression within these subclusters showed that *FCGR3A* was expressed in subclusters 0 and 1, while *NCAM1* was predominantly expressed in subcluster 2 (**Figure 7B**). This pattern may suggest a shift in NK cell composition in LC patients, with a relative decrease in the *NCAM1*-enriched subcluster (**Figure 7C**). Traditionally, CD56^+^ CD16^−^ NK cells are considered prominent cytokine producers with limited cytotoxicity, whereas CD56^dim^ CD16^+^ NK cells are known for their potent cytolytic activity but limited cytokine production (9, 39). Based on this observation, we evaluated the expression of various cytokine genes, activation markers, and cytotoxic molecules within the NK cell cluster in LC patients. This analysis revealed a decrease in the expression of *IFNG*, *NKG7*, *KLRD1*, *KLRF1*, *PRF1*, *IL2RB*, *GZMA*, *GNLY*, *NCR1*, *and BCL2* in NK cells from LC patients. Conversely, we observed an increase expression of *CD69*, *GZMB*, *LAMP1*, *KLRB1*, *RIPK1*, and *CASP8* in NK cells from the same group (**Figure 7D**). Given the mixed pattern of gene expression, some indicative of enhanced NK cell function and others of impaired activity, we calculated an activation score based on the expression of these genes. This score was significantly lower in NK cells from LC patients (**Figure 7E**). These results suggest that NK cells in LC patients not only occur at reduced frequencies but also show differences in the expression of transcripts associated with NK cell function.

### LC modifies the proportion and transcriptional profiles of B and plasma cells

**Figure 8:**
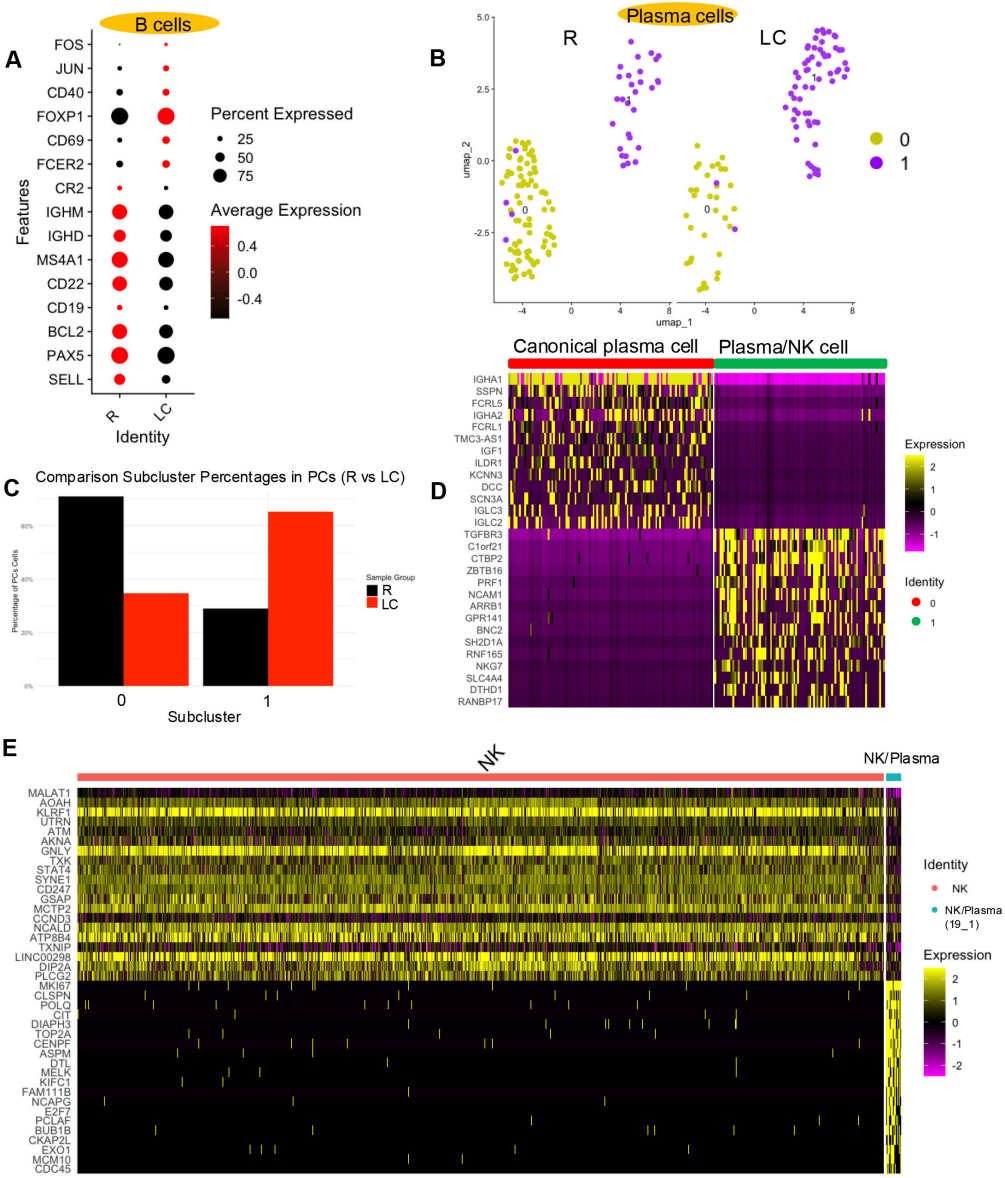
B cell and plasma cell functional gene expression and subset characterization in LC versus R individuals. **(A)** Bubble plot displaying expression patterns of B cell-associated functional genes in LC vs R cohorts. **(B)** UMAP projection showing two plasma cell (PC) subsets. **(C)** Bar plots showing the percentage distribution of PC subsets in LC and R individuals. **(D)** Heatmap displaying the top 15 upregulated and top 15 downregulated genes between two different PC subsets. **(E)** Heatmap displaying the top 20 upregulated and top 20 downregulated genes when comparing NK cells to PC subset 1.

Identification of B cells based on *CD79A* expression revealed that clusters 5, 8, and 15 correspond to B cells (**Figure 1A**, **Supplementary Figure 1H**), with clusters 5 and 15 showing expansion in LC patients (**Figure 1B**, **Supplementary Figure 3B**). To further characterize these populations, we examined the expression of genes associated with different B cell states. Genes typically associated with naïve B cells, such as *SELL*, *PAX5*, *BCL2*, *CD19*, *CD22*, *MS4A1*, *IGHD*, and *IGHM*, were upregulated in R individuals. In contrast, genes associated with effector or activated B cells, including *CR2*, *FCERs*, *CD69*, *FOXP1*, *CD40*, *JUN*, and *FOS*, were more expressed in LC patients (**Figure 8A**). These findings may suggest an increased activation state of B cells in LC patients compared to the R group.

Plasma cells (PCs) were primarily represented by cluster 19 (**Figure 1A**, **Supplementary Figure 2C**). Upon subsetting cluster 19, we identified two distinct subclusters (**Figure 8B**). The proportion of subcluster 0 was lower, while subcluster 1 was more prevalent in LC individuals (**Figure 8C**). Interestingly, subcluster 0 expressed canonical plasma cell markers such as *IGHA1*, *IGHA2*, *IGLC2*, and *IGLC3*, confirming its identity as a conventional plasma cell population. In contrast, subcluster 1 (19-1) displayed expression of several genes typically associated with NK cells (**Figure 8D**).

Given that subcluster 1 was positioned near the NK cell cluster (cluster 4) in the UMAP space (**Figure 1A**), we next questioned whether this population might represent misclassified NK cells. However, heatmap analysis revealed distinct gene expression patterns between subcluster 1 and the NK cell cluster, suggesting that despite the shared markers, subcluster 1 likely represents a unique plasma cell subset or a transitional population rather than true NK cells (**Figure 8E**).

### Activation-associated transcriptional profiles are reduced in basophils but increased in LDNs in LC patients

**Figure 9:**
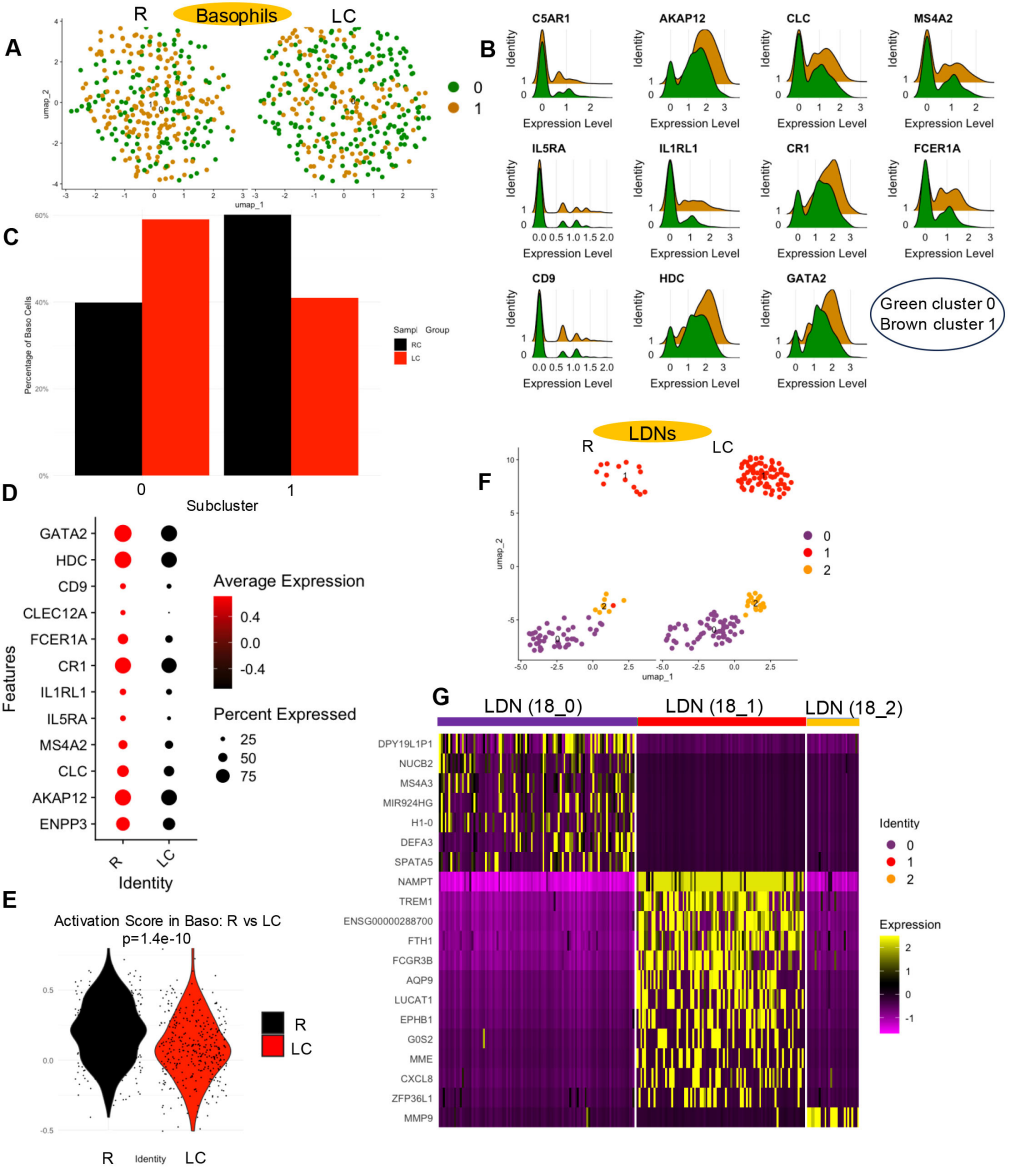
Comparative analysis of basophil activation states and LDN populations in LC versus R individuals. **(A)** UMAP projection showing two distinct basophil subsets. **(B)** Ridge plots illustrating the distribution of expression levels for basophil activity-associated genes across the two basophil subsets. **(C)** Bar plots showing the percentage distribution of basophil subsets in LC and R individuals. **(D)** Bubble plot displaying expression patterns of basophil function-associated genes in LC versus R cohorts. **(E)** Distribution of basophil activation module scores in our two different cohort. **(F)** UMAP projection of different subsets of LDNs. **(G)** Heatmap displaying differentially expressed genes in different subsets of LDNs.

When the frequency of basophils (cluster 14) in LC patients versus the R group was assessed (**Figure 1A**), no significant difference in the frequency was observed (**Figure 1B**, **Supplementary Figure 3B**). Further analysis of this population revealed the presence of two distinct subclusters (**Figure 9A**). When examining genes linked to basophil activation, such as *CD9*, *HDC*, *GATA2*, *FCER1A*, *CR1*, *IL1RL1*, *IL5RA*, *MS4A2*, *CLC*, *AKAP12*, and *C5AR1 (*40, 41*)*, we found that these genes were enriched in subcluster 1 (**Figure 9B**). Although the proportion of subcluster 0 was higher in LC patients, subcluster 1 was proportionally reduced (**Figure 9C**). We also observed a downregulation of these basophil-related activation genes in the overall basophil cluster from LC patients (**Figure 9D**). In line with these findings, the activation score derived from the expression of these genes was significantly lower in LC patients (**Figure 9E**). These findings suggest reduced activation-associated transcriptional profiles in basophils from LC patients.

We next characterized neutrophils from R and LC patients based on transcriptional profiles in our scRNA-seq dataset. Although mature neutrophils are typically excluded during Ficoll-Paque separation due to their higher density, LDNs can be found among PBMCs following leukocyte separation by density gradient centrifugation (42, 43). Analysis of Cluster 18, which corresponds to LDNs, revealed their expansion in LC patients (**Figures 1A, B**, **Supplementary Figure 3B**). Further examination of this cluster identified three subclusters, with subcluster 1 displaying a distinct gene expression profile, consistent with its separation in UMAP space (**Figure 9F**). Interestingly, only subcluster 1 was expanded in LC individuals (**Figure 9F**). A heatmap of the most highly expressed genes in each subcluster showed that subcluster 1 was enriched in genes such as *MME* and *CXCL8*, both of which are associated with neutrophil activation (**Figure 9G**). Together, these results suggest the expansion of a transcriptionally distinct LDN subset with elevated expression of activation-associated genes in LC patients.

### Functional reprogramming of monocytes and expansion of activated platelets in LC

**Figure 10:**
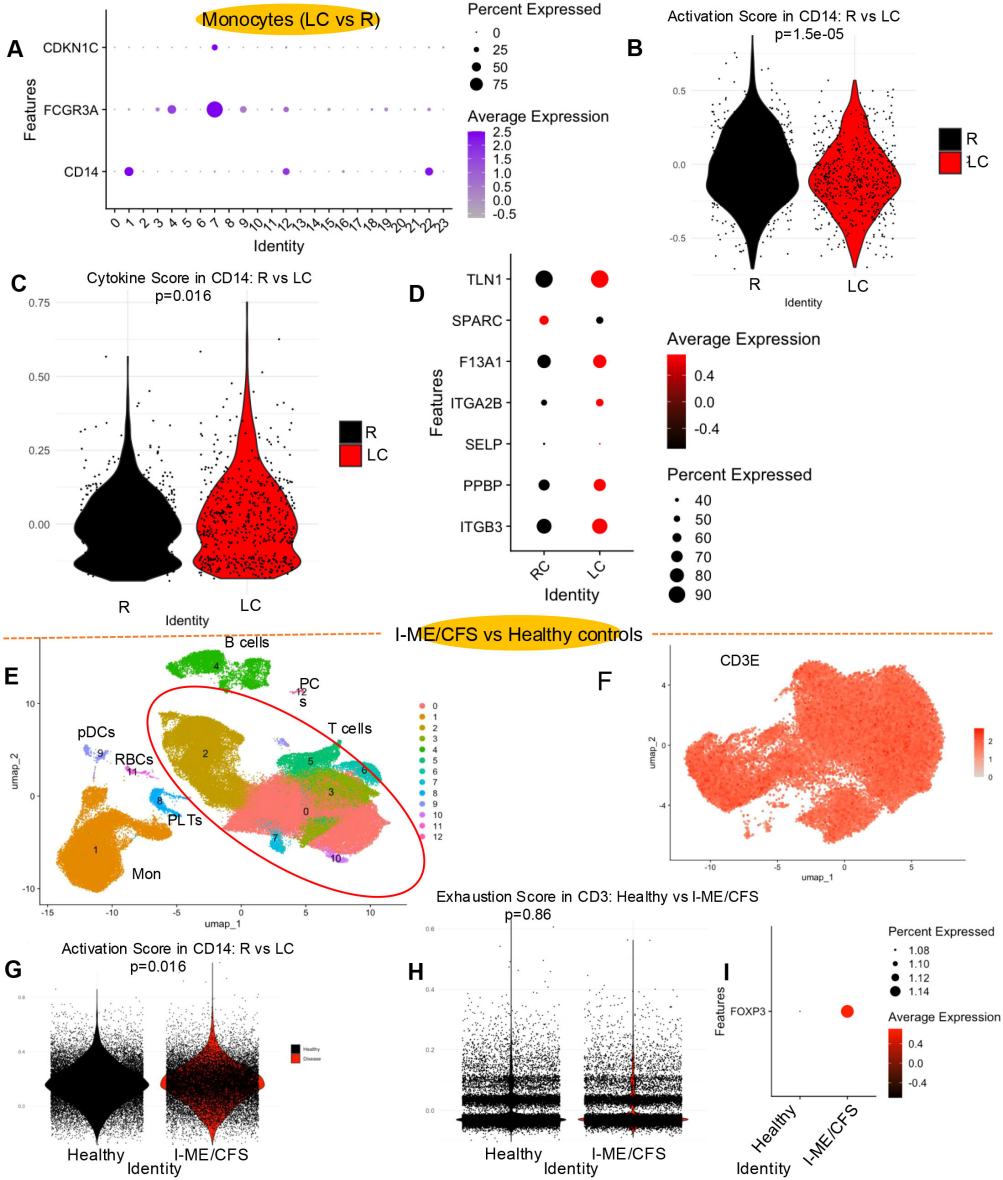
Monocyte gene expression, functional activity, and platelet-associated gene signatures in LC versus R individuals. **(A)** Bubble plot depicting the expression of monocyte-associated genes across PBMC clusters. Distribution of **(B)** monocyte phagocytic activity and **(C)** cytokine module scores in LC and R cohorts. **(D)** Bubble plot depicting differentially expressed genes associated with platelet function in LC compared to R individuals. **(E)** UMAP plot of 45,047 pooled PBMCs from 20 healthy controls and 19 idiopathic ME/CFS (I-ME/CFS) female patients. **(F)** Feature plot visualizing the spatial distribution of *CD3E*-positive cells (expression > 0.5). **(G)** Distribution of T cell activation and **(H)** exhaustion module scores in healthy individuals and patients with I-ME/CFS (Disease). **(I)** Bubble plot displaying expression pattern of *FOXP3* gene in healthy individuals and patients with I-ME/CFS.

Monocytes were classified into three distinct clusters (1, 12, and 7), corresponding to classical, intermediate, and non-classical monocytes, respectively (**Figure 1A**). This classification was based on the expression levels of key marker genes—*CD14*, *FCGR3A*, and *CDKN1C* (**Figure 10A**, **Supplementary Figure 1I**). Cluster 1 exhibited the highest expression of *CD14*, while Cluster 7 showed the highest expression of *FCGR3A* and *CDKN1C* (**Figure 10A**). Cluster 12 displayed intermediate expression of these genes and was therefore identified as the intermediate monocyte population (**Figure 10A**). Notably, there were no significant differences in the frequency of these monocyte subsets between LC patients and the R group (**Figure 1B**, **Supplementary Figure 3B**). To evaluate monocyte function, we subsetted cells with *CD14* expression levels greater than 0.5 and assessed the expression of genes associated with phagocytosis (*TLR1, TLR2, TLR3, TLR4, TLR8, CD80*, and *CD86*) and cytokine signaling (*IL1B, TNF, CXCL8, IL23A, IL12A, IL27*, and *IL15*). The phagocytosis-associated gene score was reduced (**Figure 10B**), whereas a cytokine-associated gene score was elevated in monocytes from LC patients (**Figure 10C**). These findings reflect a shift in transcriptional profile of monocytes in LC patients, characterized by reduced expression of phagocytic-related genes but enhanced expression of pro-inflammatory cytokine genes.

Finally, we analyzed platelets assigned to cluster 21 and compared their distribution between recovered (R) individuals and LC patients. As shown in **Figure 1A** and **Supplementary Figure 3B**, platelets were markedly expanded in LC patients. Further subclustering of this population revealed two distinct platelet subclusters, both of which were also expanded in the LC group (**Supplementary Figure 6B**). In addition, genes associated with platelet activation were upregulated in platelets from LC patients (**Figure 10D**).

### Comparison of healthy controls to idiopathic ME/CFS patients

To determine whether the immune cell changes observed in ME/CFS associated with LC are also present in idiopathic ME/CFS, we re-analyzed an independent, publicly available scRNA-seq dataset comparing PBMC gene expression profiles from individuals with ME/CFS and healthy controls (19). To ensure consistency with our dataset, we included only female participants, comprising 20 healthy controls and 19 ME/CFS patients. All samples were collected prior to the COVID-19 pandemic, ruling out the possibility of LC misclassification. After quality control, 45,047 cells were retained for downstream analysis. UMAP clustering identified 13 distinct cell clusters based on the expression of canonical marker genes (**Figure 10E**, **Supplementary Figure 7A**). In addition to the previously described markers, we used *CLEC4C* and *SCT* to define plasmacytoid dendritic cells (pDCs; cluster 9), and *HBA1* and *HBA2* to identify red blood cells (RBCs; cluster 11) (**Figure 10E**, **Supplementary Figure 7A**).

We merged the cells from two conditions and evaluated the function of different immune cells (**Supplementary Figure 7B**). Cells with *CD3E* expression greater than 0.5 were selected as T cells (**Figure 10F**), and their activation status was assessed using a module score based on the following genes: *IFNG*, *GZMB*, *PRF1*, *CTLA4*, *FOS*, *TNF*, *IL2RA*, *EOMES*, *TOX*, *IL2*, *CD44*, *MYC*, *TBX21*, *ICOS*, *CD69*, and *JUN*. This analysis revealed higher T cell activation in ME/CFS patients compared to healthy controls (**Figure 10G**). In contrast, no significant difference in T cell exhaustion status was observed between groups when assessed using expression of exhaustion-associated genes: *CTLA4*, *EOMES*, *TOX*, *TIGIT*, *PDCD1*, *LAG3*, *HAVCR2*, *CD160*, *ENTPD1*, and *BATF* (**Figure 10H**). Notably, unlike our cohort, *FOXP3* expression was upregulated in idiopathic ME/CFS patients (**Figure 10I**).

We were unable to identify a distinct cluster corresponding specifically to MAIT cells or NK cells using canonical markers in this dataset. However, cluster 2 exhibited high expression of genes associated with MAIT cells, γδ T cells, and NK cells (**Supplementary Figure 7C**). We therefore subsetted cluster 2 and found that subclusters 0 and 3 showed elevated expression of MAIT cell–related genes, including *ZBTB16* and *IL18RAP*, and also included NK cells, as indicated by low expression of *CD4*, *CD8A*, and *CD8B*, and high expression of NK cell markers such as *KLRD1*, *NCAM1*, *NCR1*, *FCGR3A*, *KLRK1*, *PRF1*, *GZMB*, and *GNLY* (**Supplementary Figures 7D, E**). Based on these findings, we calculated activation scores for both MAIT and NK cells within these subclusters. This analysis revealed no significant difference in NK cell activation between healthy individuals and ME/CFS patients (**Supplementary Figure 7F**). However, we observed a higher activation score in MAIT cells from ME/CFS patients compared to healthy controls (**Supplementary Figure 7G**) without any changes in the proportion of subclusters 0 and 3 between groups (**Supplementary Figure 8A**).

We next assessed the function of B cells by analyzing the expression of genes associated with naïve and activated B cell subsets, using the same gene set applied in our dataset. We observed both upregulation and downregulation of genes corresponding to different B cell subsets, suggesting a dysregulated distribution of B cell types between ME/CFS patients and healthy individuals (**Supplementary Figure 8B**). Additionally, platelet-related gene expression in ME/CFS patients showed mixed patterns of upregulation and downregulation, indicating alterations in platelet activation status (**Supplementary Figure 8C**). In contrast to our LC/ME/CFS cohort, we found no significant differences in activation or cytokine scores in monocytes (cluster 1) between the two groups (**Supplementary Figures 8D, E**). We also evaluated the activation status of pDCs by examining the expression of genes associated with their activation, including *IRF7*, *IRF3*, *IRF4*, *TLR7*, *TLR9*, *ISG15*, *MX1*, *IFI6*, *IFI44L*, *IFIT1*, *IFIT3*, *OAS1*, *OAS2*, *OASL*, *TNFSF10*, *IL6*, *CD86*, *CD83*, *HLA-DRA*, *HLA-DRB1*, *LAMP3*, *BST2*, *LY6E*, and *CLEC4C*. This analysis revealed upregulation of certain genes, such as *CLEC4C* and *LY6E*, and downregulation of others, including *HLA-DRA* (**Supplementary Figure 8F**). However, the overall activation score of pDCs did not differ significantly between ME/CFS patients and healthy controls (**Supplementary Figure 8G**).

### Gal-9, through interaction with TIM-3, promotes MAIT cell depletion in LC patients

**Figure 11:**
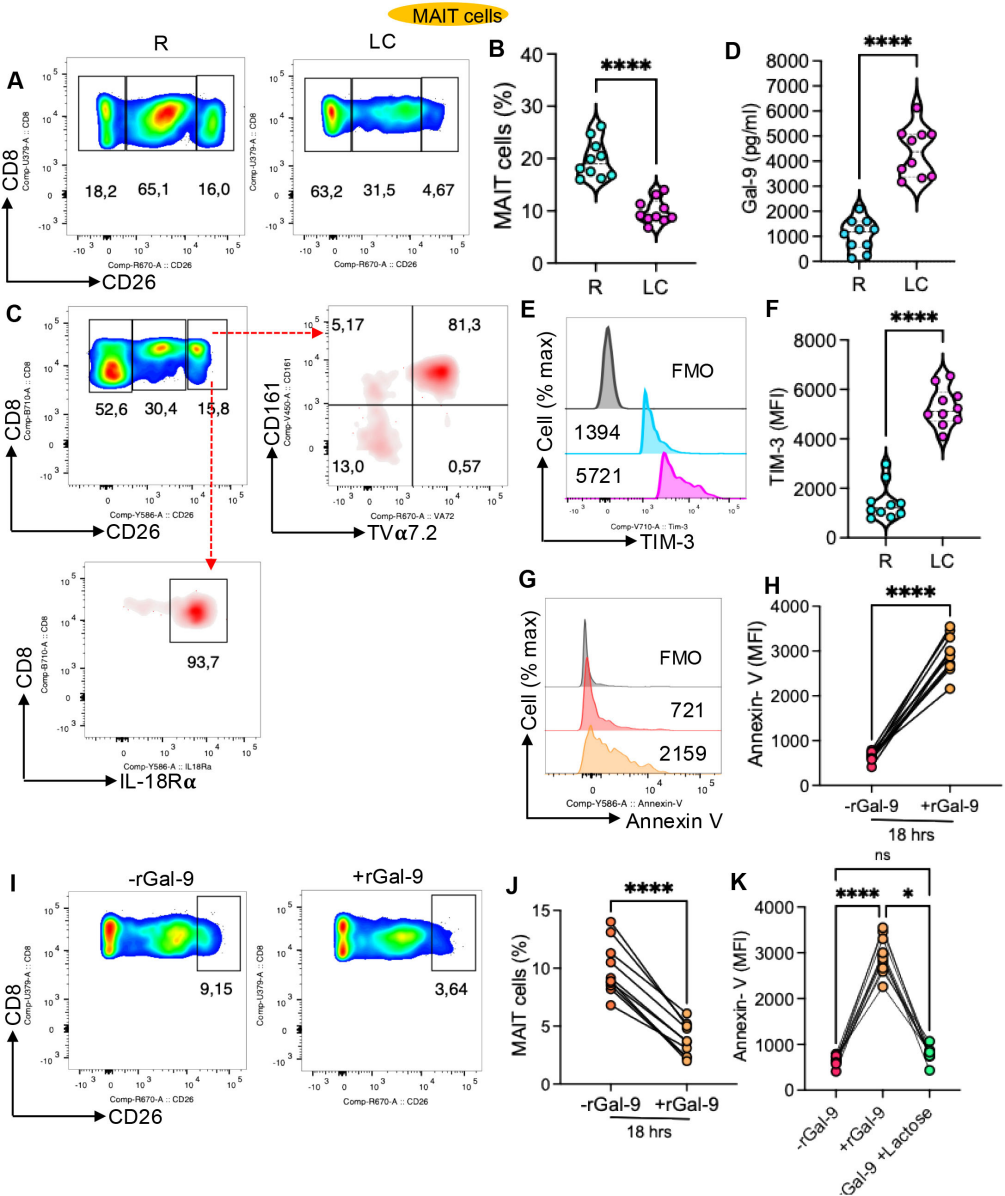
Gal-9 promotes apoptosis of MAIT cells from LC patients. **(A)** Representative flow cytometry plots and **(B)** cumulative data showing the frequency of CD8^+^CD26^hi^ T cells in PBMCs from R and LC patients. **(C)** Representative plots of expressed MAIT cell markers in CD8^+^CD26^hi^ T cells. **(D)** Plasma concentrations of Gal-9 in R and LC patients. **(E)** Representative histogram plots and **(F)** cumulative data showing TIM-3 expression (measured by mean fluorescence intensity; MFI) in MAIT cells from R and LC patients. **(G)** Representative histogram plots and **(H)** cumulative data of Annexin-V expression in MAIT cells within PBMCs of LC patients cultured without recombinant Gal-9 (-rGal-9) or with rGal-9 (+Gal-9; 0.5 μg/ml overnight). **(I)** Representative flow cytometry plots and **(J)** cumulative data showing the proportion of MAIT cells in the absence or presence of rGal-9 (0.5 μg/ml overnight). **(K)** Cumulative data of apoptotic MAIT cells cultured in the absence or presence of rGal-9, with or without lactose (30 mM). *P*-values were calculated using a two tailed Mann–Whitney U test **(B, D, F)** and Wilcoxon matched-pairs signed-rank tests **(H, J, K)**; *P< 0.05, ****< 0.0001. Each dot represents an individual study subject. Blue and pink reflect R and LC, respectively.

In agreement with our scRNAseq data in the present study, we observed a significant reduction in the frequency of CD8^+^CD26^hi^ T cells (**Figures 11A, B**), which express MAIT-associated markers CD161, TCR Vα7.2, and IL-18Rα (**Figure 11C**, **Supplementary Figure 8H**) (44). Consistent with our previous findings (2), plasma Gal-9 levels were elevated in LC patients compared to the R group (**Figure 11D**). Given the activation state of MAIT cells (**Figure 6E**), we found that these cells exhibited significantly higher levels of TIM-3 expression than their counterparts in the R group (**Figures 11E, F**). Since Gal-9 is known to induce apoptosis and suppress TIM-3+ T cells (7, 45), we examined its effect on MAIT cells using total PBMCs. These experiments revealed that recombinant Gal-9 (rGal-9) enhanced MAIT cell apoptosis (**Figures 10G, H**), which subsequently resulted in a reduction in MAIT cells (**Figures 11I, J**). Notably, the addition of lactose (30 mM) (46) partially but significantly reversed rGal-9-induced MAIT cell apoptosis (**Figure 11K**), consistent with previous reports in other models (3, 44).

### Gal-9, through interaction with TIM-3, promotes γδ T cell depletion in LC patients

**Figure 12:**
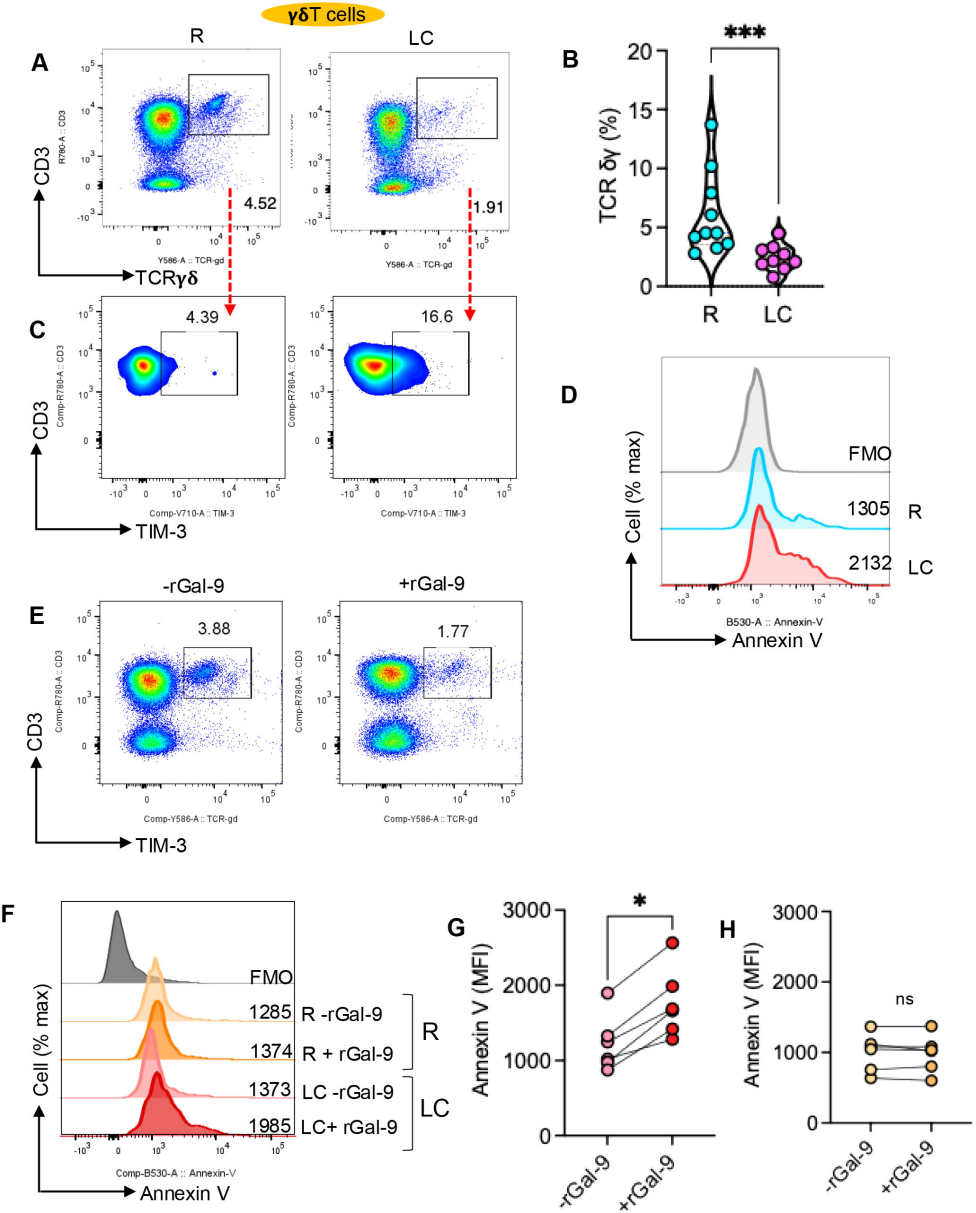
Gal-9 promotes apoptosis of γδ T cells from LC patients. **(A)** Representative flow cytometry plots and **(B)** cumulative data showing the frequency of γδ T cells in PBMCs from R and LC patients. **(C)** Representative flow cytometry plots of TIM-3 expression in γδ T cells from R and LC patients. **(D)** Representative histogram plots of Annexin-V expression in γδ T cells from R and LC patients. **(E)** Representative flow cytometry plots of percentages of γδ T cells in PBMCs from a LC individual in the absence or presence of rGal-9 (0.5 μg/ml overnight). **(F)** Representative histogram plots, and **(G)** cumulative data of apoptotic γδ T cells cultured in the absence or presence of rGal-9 overnight from LC patients or **(H)** the R group. *P*-values were calculated using a two tailed Mann–Whitney U test **(B)** and Wilcoxon matched-pairs signed-rank tests **(G, H)**; *P< 0.05, ***< 0.001. Each dot represents an individual study subject. Not significant (ns).

We also found a significant reduction in the frequency of γδ T cells in LC patients (**Figures 12A, B**). Similar to MAIT cells, γδ T cells in LC patients exhibited elevated TIM-3 expression (**Figure 12C**). Notably, increased apoptotic potential of γδ T cells in LC patients was evident in freshly isolated cells (**Figure 12D**). This was further confirmed by *in vitro* experiments, where overnight culture of PBMCs with rGal-9 promoted γδ T cells depletion via enhanced apoptosis (**Figures 12E–G**). However, this effect was not observed in γδ T cells from the R group (**Figures 12F, H**). Collectively, these observations provide a potential mechanistic explanation for MAIT and γδ T cell activation and apoptosis in LC patients.

## Discussion

This study provides a high-resolution single-cell transcriptomic landscape of peripheral immune cells in LC patients with ME/CFS. Our findings support and extend our previous reports and reveal distinct immunological signatures that may underpin the persistent symptoms observed in LC-ME/CFS patients.

In this study, we observed a reduction in naïve T cells (both CD4^+^ and CD8^+^), accompanied by an expansion of effector T cell subsets and higher exhaustion scores in LC patients. These shifts in T cell composition reflect ongoing immune activation and are consistent with previous reports suggesting chronic immune dysregulation, sustained T cell turnover, and T cell exhaustion in LC patients (3, 5, 10). Notably, effector T cells displayed heightened expression of pro-inflammatory cytokines, granzyme B (*GZMB*), and perforin (*PRF1*), including ongoing immune activation. Such persistent immune activation may contribute to tissue inflammation, neural dysfunction, and the systemic symptoms observed in LC (10, 11). Elevated Gal-9 may originate from activated neutrophils (42) as well as other immune and non-immune cells, acting as a damage-associated molecular pattern (DAMP) that influences both innate and adaptive immune cells (47). In particular, Gal-9 may further sustain immune activation and T cell stimulation through interactions with the TCR (8, 48), thereby promoting T cell exhaustion (7, 49), as reported in other pathological conditions (8, 50). The reduction in Tregs, combined with the increased effector T cells, could contribute to immune dysregulation and sustained inflammation in LC patients, a hallmark of the disease (5, 10, 11). Although our scRNAseq analyses identify distinct transcriptional states across immune cell populations in LC, these data primarily reflect gene expression programs rather than direct cellular function. Accordingly, functional interpretations should be made with caution and ideally supported by orthogonal assays. However, our previous study in the same cohort demonstrated functional immune dysregulation and T cells exhaustion (3), which has been observed in memory CD8 T cells in idiopathic ME/CFS (51).

Consistent with recent systems-level multi-omics analyses of idiopathic ME/CFS (52), our scRNA-seq data suggest that γδ T cells and MAIT cells in ME/CFS are transcriptionally skewed toward a pro-inflammatory effector state rather than a classical hypo-functional or exhausted phenotype. Specifically, these populations exhibit enrichment of inflammatory programs associated with IFN-γ and cytotoxic effector molecules, supporting the notion that mucosal-associated innate-like T cells may contribute to sustained inflammatory tone in ME/CFS. Nevertheless, together with prior studies linking γδ T and MAIT cell activation to gut microbial and metabolic perturbations (52), our findings support a model in which altered mucosal immune–metabolic crosstalk, rather than intrinsic lymphocyte dysfunction, underlies the immune dysregulation observed in idiopathic ME/CFS and LC patients with ME/CFS. Thus, the loss of polyfunctional MAIT cells could compromise the immune system’s ability to respond to secondary infections (44). Similarly, the decrease in γδ T cells, which play critical roles in mucosal immunity and tissue repair, may contribute to immune dysregulation in LC, particularly within gut and epithelial compartments where microbial translocation has been implicated (2, 53). Although the mechanisms underlying MAIT and γδ T cell deficiency in LC remain unclear, our findings demonstrate that elevated Gal-9 promotes apoptosis and depletion of these cells through interaction with TIM-3, contributing to broader immune dysregulation and the loss of key antiviral effector populations (54).

Our analysis also revealed reduced frequencies and altered transcriptional profile of NK cells in LC patients (11). While a subset of NK cells showed elevated expression of activation-associated transcripts, the overall NK cell activation score was reduced, indicating a dysregulated phenotype. Although sample limitations prevented us from directly assessing the effects of Gal-9 on NK cells, previous studies have reported that Gal-9 is associated with NK cell dysregulation in HIV and cancer (9, 50). Therefore, it is plausible that elevated Gal-9 may similarly impair NK cell effector functions in LC patients. Further analysis revealed a selective reduction in CD56^+^ NK cells, which are known for their cytokine production (39). This may imply an altered NK cell subset composition and activation profiles that could lead to the persistent immune dysregulation observed in LC patients. However, the mixed gene expression profile of NK cells in LC patients warrants further functional studies to determine whether their cytotoxic potential is ultimately diminished as reported in idiopathic ME/CFS (55). Several studies have reported dysfunctional NK cells with impaired degranulation capacity and reduced cytotoxicity in patients with idiopathic ME/CSF patients (55–57). In particular, dysregulation of mitochondrial energy-generating pathways in NK cells has been described and may contribute to NK cell dysfunction in idiopathic ME/CFS patients (58). More recently, genetic studies have implicated NK cells in idiopathic ME/CFS, showing that specific alleles of genes encoding inhibitory KIR receptors (*KIR3DL3*, *KIR3DL2* and *KIR3DL1)* may be associated with susceptibility to ME/CFS (59).

The upregulation of select activation-associated transcripts in B cells suggests a shift toward an activated and dysregulated phenotype as seen in acute COVID-19 (60). These transcriptional changes could contribute to autoimmune or chronic inflammatory processes reported in LC (3). B cell and plasma cell populations also displayed distinct shifts. B cells in LC patients showed increased expression of activation-related genes (e.g. *CD69*, *JUN*, and *FOS*), while plasma cells included a subpopulation co-expressing NK-related markers, suggesting potential transitional or dysregulated states. However, this observation warrants further investigation into the relationship between plasma cell differentiation and NK cells in LC. The upregulation of hormone receptor genes (ESR1, ESR2, AR, and NR3C1) in B cell subsets of female LC patients further supports the hypothesis that hormonal signaling pathways may influence autoantibody production (3) and immune dysregulation in LC (61), particularly in women who are disproportionately affected by both LC and ME/CFS (3, 62).

Monocyte populations, although numerically unchanged, exhibited reduced expression of phagocytic-associated genes and increased expression of pro-inflammatory cytokine genes, suggesting a phenotypic shift toward an inflammatory monocyte state. This skewed monocyte function likely contributes to the systemic inflammation seen in LC, as supported by elevated CRP and SAA levels previously reported in LC cohorts (3). Additionally, LDNs were expanded and exhibited activation signatures, a finding that aligns with recent studies suggesting that neutrophil dysregulation may play a role in LC pathogenesis (63). Although mature neutrophils were not subjected to scRNAseq, we have previously reported their expansion in LC patients. In particular, we observed the abundance of a highly activated neutrophil phenotype (33), which by shedding Gal-9 contribute to the elevation of plasma Gal-9 and subsequently MAIT apoptosis and chronic immune activation in LC patients (3, 47, 64).

Moreover, platelets were notably expanded in LC patients and exhibited elevated expression of genes linked to platelet activation. These findings are consistent with emerging evidence implicating platelet dysfunction in LC and raise the possibility of ongoing vascular inflammation or coagulopathy contributing to systemic symptoms (63, 65).

Upregulated genes such as RELN, RPL17-C18orf32, ZFHX3, and ZNF469 were detected across various immune cell types, providing insight into the cellular sources of these previously identified biomarkers in LC-ME/CFS (11). These findings suggest that the immune dysregulation observed in LC may be driven by changes in both innate and adaptive immune responses. The upregulation of genes such as ZFHX3 and ZNF469 in monocytes and DCs could be indicative of an important role for these genes in myeloid cells. Additionally, these genes are involved in neuronal differentiation (66, 67) and extracellular matrix remodeling (68), as such their upregulation may contribute to LC pathogenesis.

Of particular interest, *RELN*, which encodes the extracellular matrix protein Reelin, was consistently elevated in LC patients (11). Given its dual roles in neurodevelopment and immune regulation (18, 69), Reelin may represent a mechanistic link between neuroinflammation and cognitive symptoms in LC, such as brain fog and impaired memory. Its expression in both immune and neuronal contexts warrants further investigation as a potential biomarker and therapeutic target. These findings not only provide novel insight into the specific source of each upregulated gene but also highlight the non-PBMC source of other undetected genes in our whole blood bulk RNAseq, such as *FEZF2*, *BRINP2*, *HOXC12*, *SKOR2*, and *EGR3 (*11).

While bulk RNA-seq revealed modulation of multiple transcripts that were not detected within immune cell populations captured by scRNA-seq, these differences likely reflect the distinct cellular and molecular compartments interrogated by each platform. Whole-blood transcriptomic profiling captures contributions from granulocytes, platelets, and erythroid-lineage cells, as well as cell-free and vesicle-associated RNA derived from non-immune somatic tissues. In contrast, PBMC-based scRNAseq is inherently restricted to mononuclear immune populations. Thus, platform- and compartment-specific differences should be interpreted as complementary rather than discordant, collectively providing a more comprehensive view of systemic transcriptional alterations in LC.

In contrast to the marked immune dysregulation observed in LC-ME/CFS patients, our comparative analysis of immune cells in idiopathic ME/CFS patients revealed a less pronounced and subtler shift in immune cell composition and function. While we did observe some degree of T cell activation in idiopathic ME/CFS patients, this activation was not associated with T cell exhaustion seen in LC-ME/CFS. Notably, FOXP3 was upregulated in the idiopathic group, which contrasts with the reduced Treg frequency observed in LC patients. Despite these differences, certain similarities were found between the two patient groups. MAIT cells in idiopathic ME/CFS patients also exhibited an activated phenotype, akin to the activation observed in LC-ME/CFS patients. This suggests that, in both conditions, MAIT cells may be involved in a compensatory immune response or may exhibit skewed differentiation, potentially as part of a broader immune system dysregulation.

Interestingly, no significant changes in the frequency of NK cells were observed in the idiopathic group, a finding that contrasts with the reduced NK cell activation and altered subset composition observed in LC patients. This suggests that NK cell dysfunction may be a more specific feature of LC-ME/CFS, potentially contributing to the persistent immune activation seen in these patients. In LC-ME/CFS, B cells displayed robust activation-related changes, consistent with chronic immune activation. In contrast, B cells from idiopathic ME/CFS patients exhibited a more dysregulated phenotype, without the clear activation signature seen in our LC group. This may point to distinct underlying mechanisms in immune dysregulation between the two subtypes of ME/CFS. Similarly, while platelets in LC-ME/CFS patients showed clear activation profiles, platelets in idiopathic ME/CFS patients demonstrated gene signatures indicative of dysfunction.

Overall, our findings suggest that while both LC-ME/CFS and idiopathic ME/CFS share some immune system abnormalities, the immune landscapes of the two groups diverge in several important aspects, particularly regarding T cell exhaustion, NK cell dysfunction, and the overall activation status of B cells and platelets. These differences underscore the need for a more nuanced understanding of the immunological underpinnings of these two subtypes of ME/CFS, which may ultimately inform more targeted therapeutic strategies.

In summary, this study provides insight into the immune landscape of LC with ME/CFS, revealing widespread transcriptional remodeling across both innate and adaptive immune compartments. LC was characterized by enrichment of interferon-associated signaling, TNFα/NFκB-mediated inflammation, complement pathways, and metabolic alterations, consistent with persistent immune activation. These signatures were most prominent in monocytes and LDNs, highlighting a central role for innate immune dysregulation. In parallel, multiple lymphoid populations, including CD4^+^ and CD8^+^ T cells, MAIT cells, γδ T cells, and NK cells, exhibited interferon-dominated transcriptional programs, suggestive of sustained cytokine exposure rather than acute antiviral responses (16). Notably, plasma cells displayed reduced oxidative phosphorylation–related pathways, indicating altered metabolic fitness within humoral compartments. Collectively, these findings support a model in which LC with ME/CFS is associated with chronic immune activation involving coordinated remodeling of innate and adaptive immune pathways, providing a transcriptional framework that may contribute to disease persistence and inform future biomarker and therapeutic development.

LC with ME/CFS disproportionately affects women compared to men (20); accordingly, the present study focused exclusively on female participants. While this approach reduces biological heterogeneity, future studies incorporating both sexes will be necessary to elucidate potential sex-specific differences in disease pathophysiology. In addition, larger cohorts with longitudinal sampling, together with integration of complementary proteomic and metabolomic datasets, will be essential to validate these findings and to further resolve the complex interplay between immune, endocrine, and neurological systems in LC-associated ME/CFS. Extending single-cell analyses to tissue-specific compartments and cerebrospinal fluid will also be critical for capturing both systemic and local immune dysregulation.

Although LC and idiopathic ME/CFS are increasingly discussed as overlapping infection-associated chronic illness phenotypes, distinguishing shared chronic biology from effects related to time since infection remains a key challenge. Longitudinal follow-up and direct comparisons with pre-pandemic ME/CFS cohorts matched for illness duration will be required to disentangle time-since-infection–dependent immune alterations from convergent chronic disease mechanisms.

Finally, it is important to emphasize that scRNA-seq–derived changes in cell abundance and transcriptional programs reflect immune cell states rather than direct functional capacity, particularly for sparse populations (e.g. MAIT and γδ T cells) and in the context of compositional shifts. Future studies incorporating orthogonal functional assays will be necessary to determine how these transcriptional alterations translate into immune effector function in LC-associated ME/CFS.

## Data Availability

RNAseq data are publicly available at the SRA portal of NCBI under the Accession Number PRJNA1257454.
