# Supplementary material for: Single-cell analysis reveals immune remodeling of monocytes, NK cells, T cell exhaustion, and Galectin-9–associated depletion of gamma delta and mucosal-associated invariant T cells in Long COVID with ME/CFS
Source: Front Immunol. 2026 Feb 25;17:1745933. doi: 10.3389/fimmu.2026.1745933 (PMC12975919; doi:10.3389/fimmu.2026.1745933)
Supplement: Supplementary file 1 [file DataSheet1.pdf]

S Fig 1

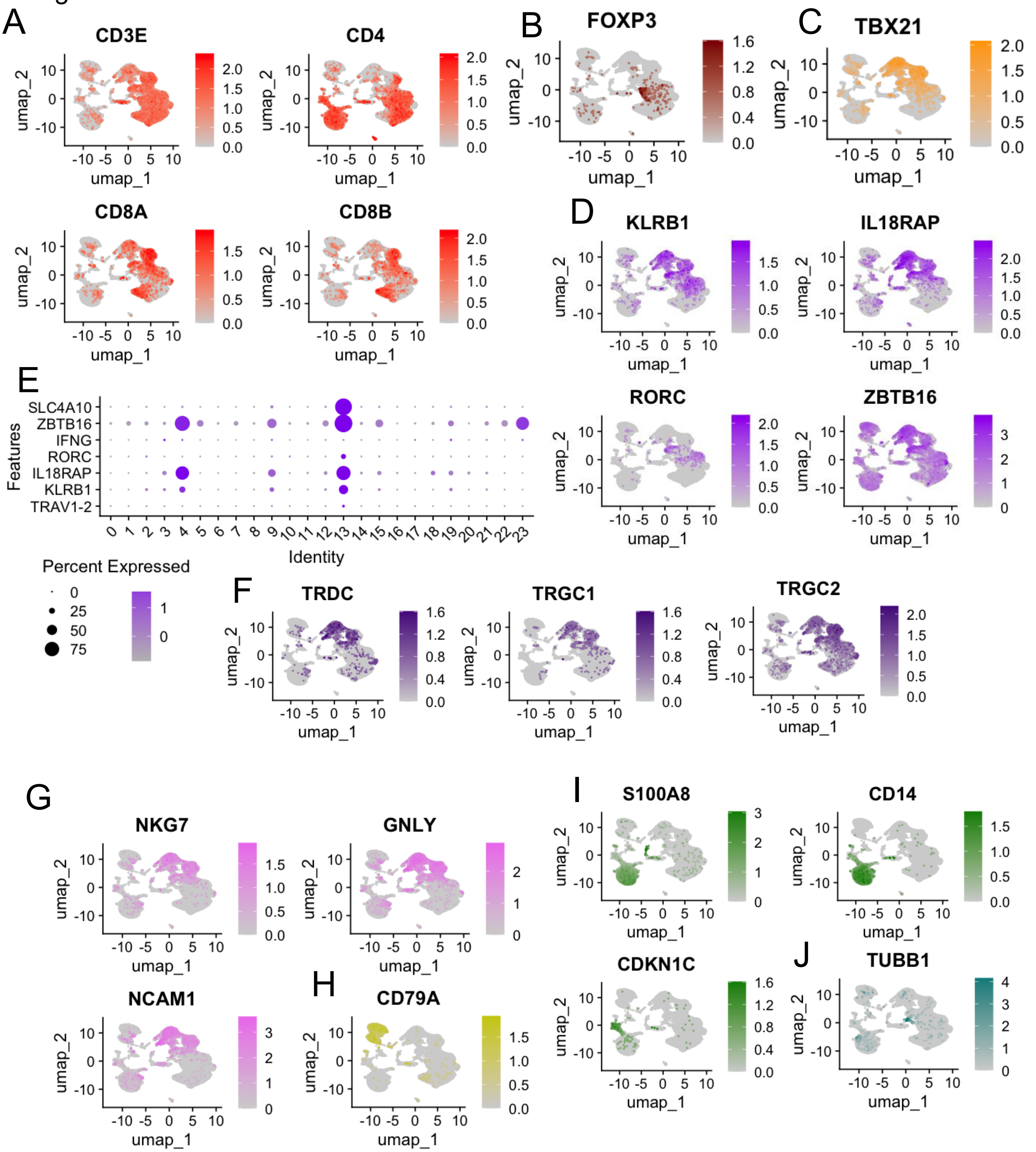

Fig S1. Feature plots showing the expression of the canonical genes associated with (**A**) T cell, (**B**) Tregs, (**C**) CTLs, and (**D**) MAIT cells. (**E**) Bubble plot illustrating the expression of the genes associated with MAIT cells across different clusters of PBCMs. Feature plots showing the expression of the canonical genes associated with (**F**) γδ T cells, (**G**) NK cells, (**H**) B cells, (**I**) monocytes and (**J**) platelets.

S Fig 2

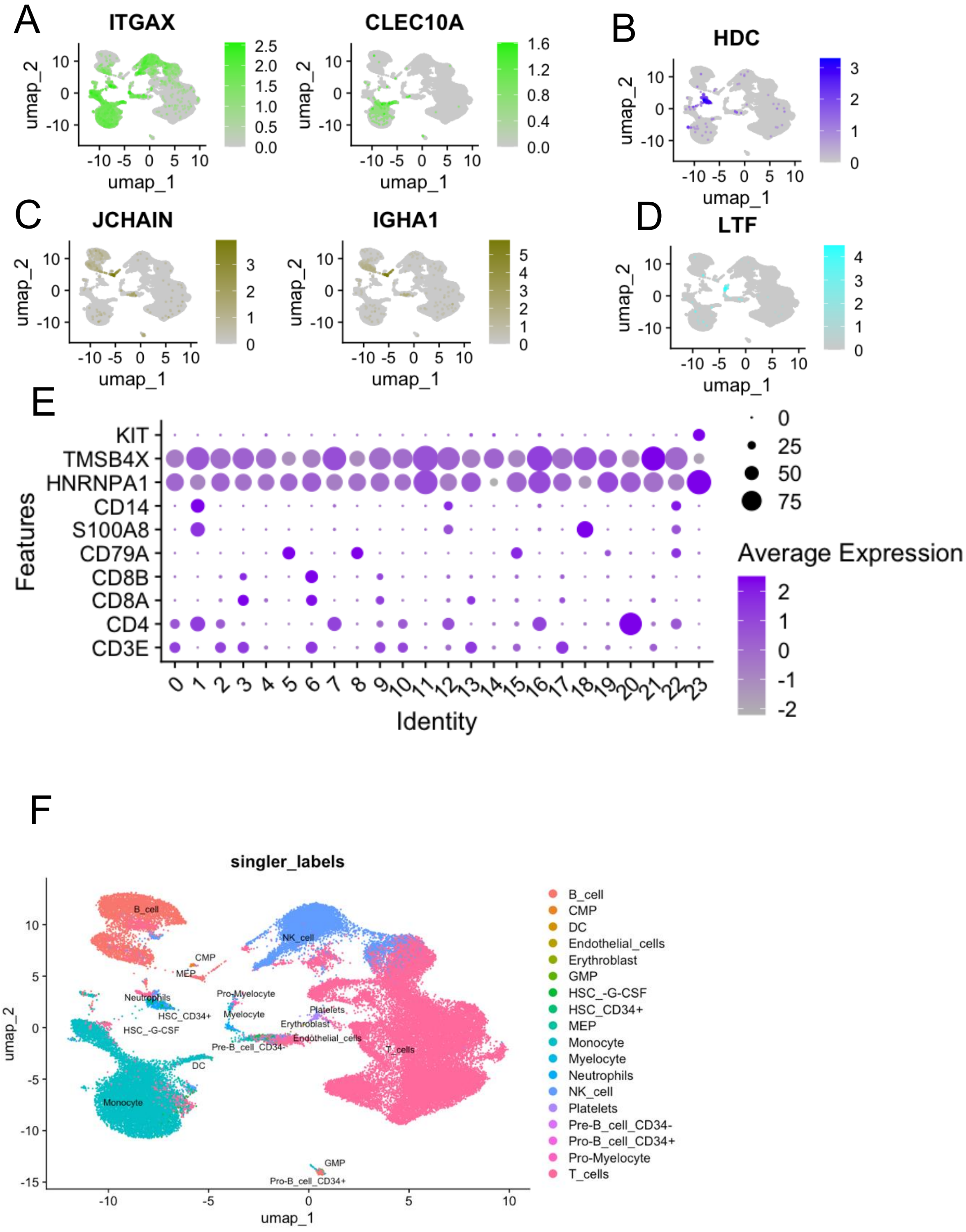

Fig S2. Feature plots showing the expression of the genes associated with (**A**) dendritic cells, (**B**) basophils, (**C**) plasma cells, and (**D**) LDNs. (**E**) Bubble plot illustrating the expression of the genes associated with innate-like lymphocytes and progenitor cells across different clusters of PBCMs. (**F**) Annotation of PBMC clusters using SingleR.

S Fig 3

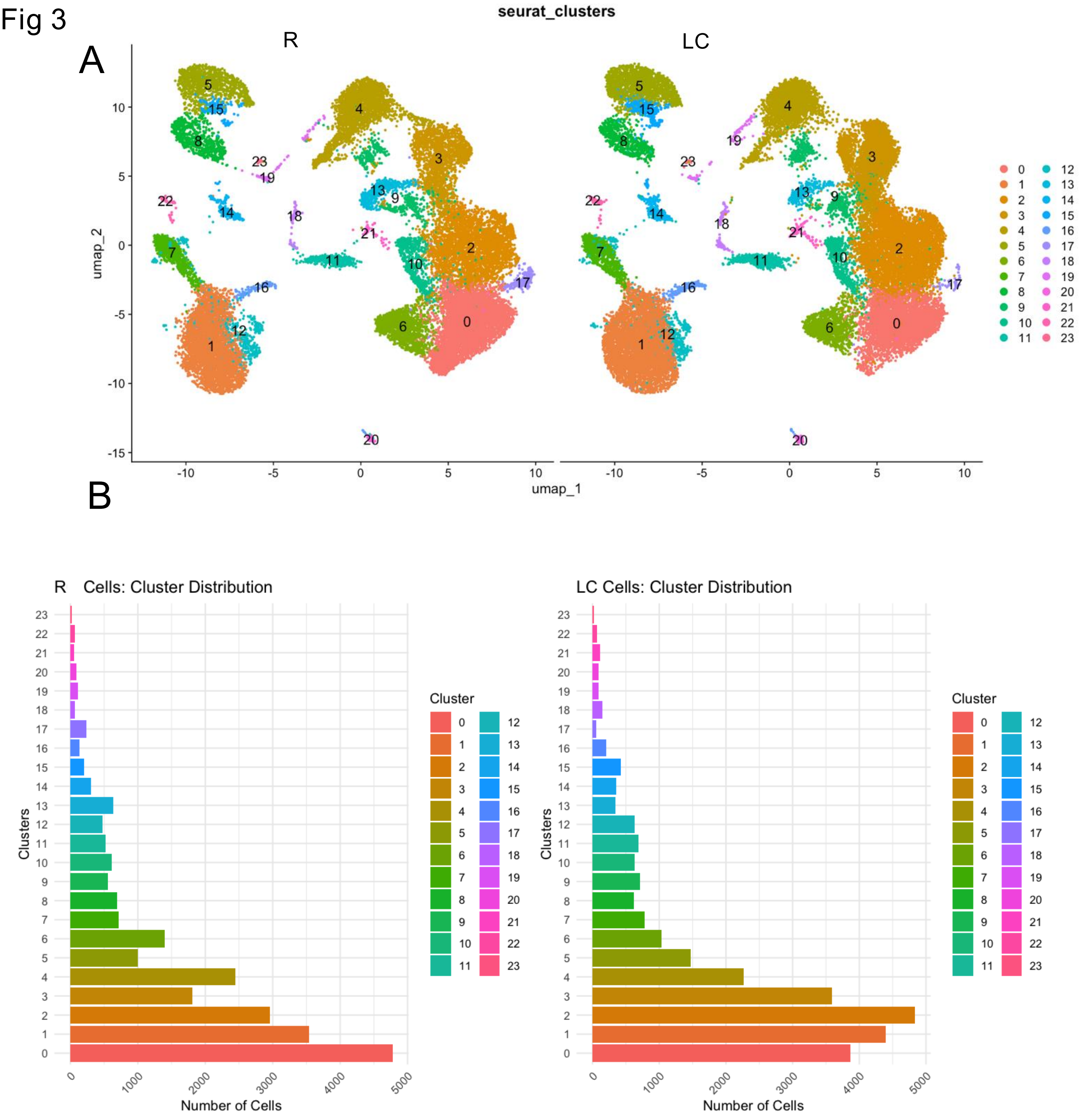

Fig S3. (**A**) UMAP plot of merged PBMCs from LC and R patients. (**B**) Bar plot showing the count of global immune cell clusters in PBMCs from our two different cohorts.

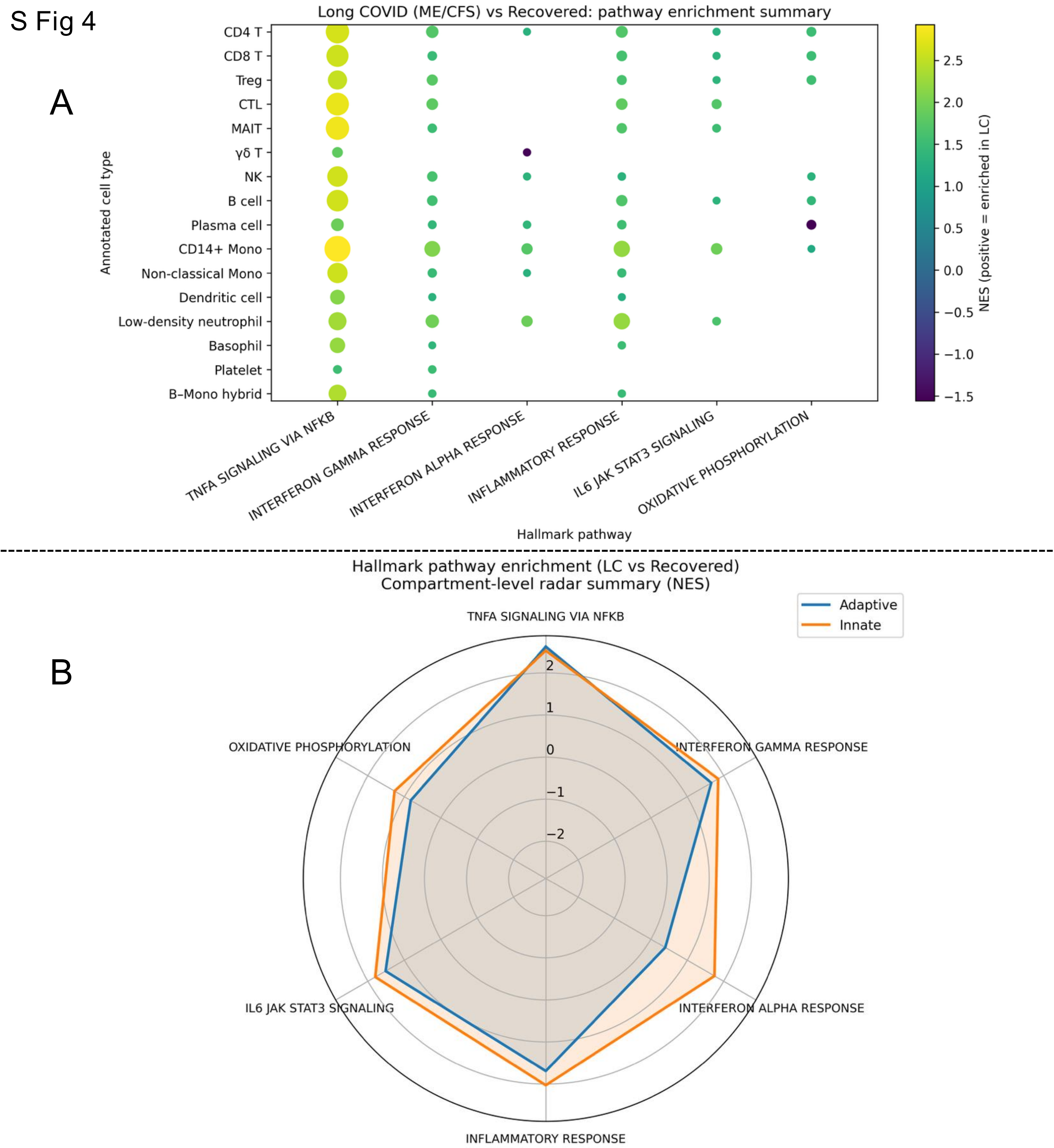

Fig. S4. (A) Pathway enrichment across immune cell subsets in LC with ME/CFS. Hallmark pathway enrichment was assessed by GSEA for each annotated cell type. Dot color indicates normalized enrichment score (NES; positive = enriched in LC), and dot size represents –log10 adjusted p value. (**B)** Radar plot summarizing Hallmark pathway enrichment across adaptive and innate immune compartments. Each axis represents a selected Hallmark pathway, and polygon distance from the center reflects NES magnitude, with positive values indicating enrichment in LC. Adaptive and innate compartments represent aggregated transcriptional programs across corresponding immune cell subsets.

S Fig 5

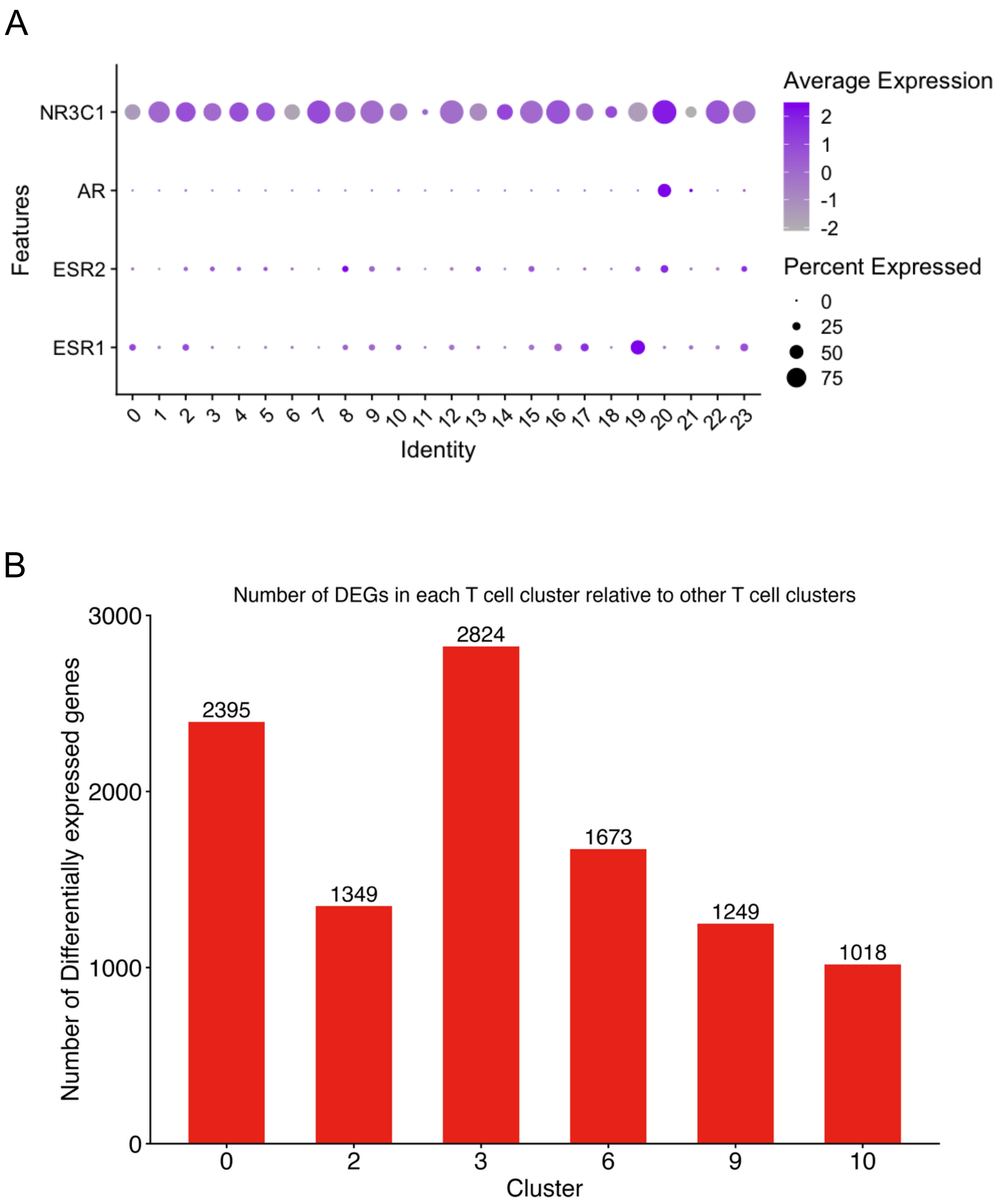

Fig. S5. (A) Bubble plot illustrating the expression of the genes associated with hormone-related receptor genes across different PBMC clusters. (**B**) Showing the number of differentially expressed genes among different T cell subsets in LC patients.

S Fig 6

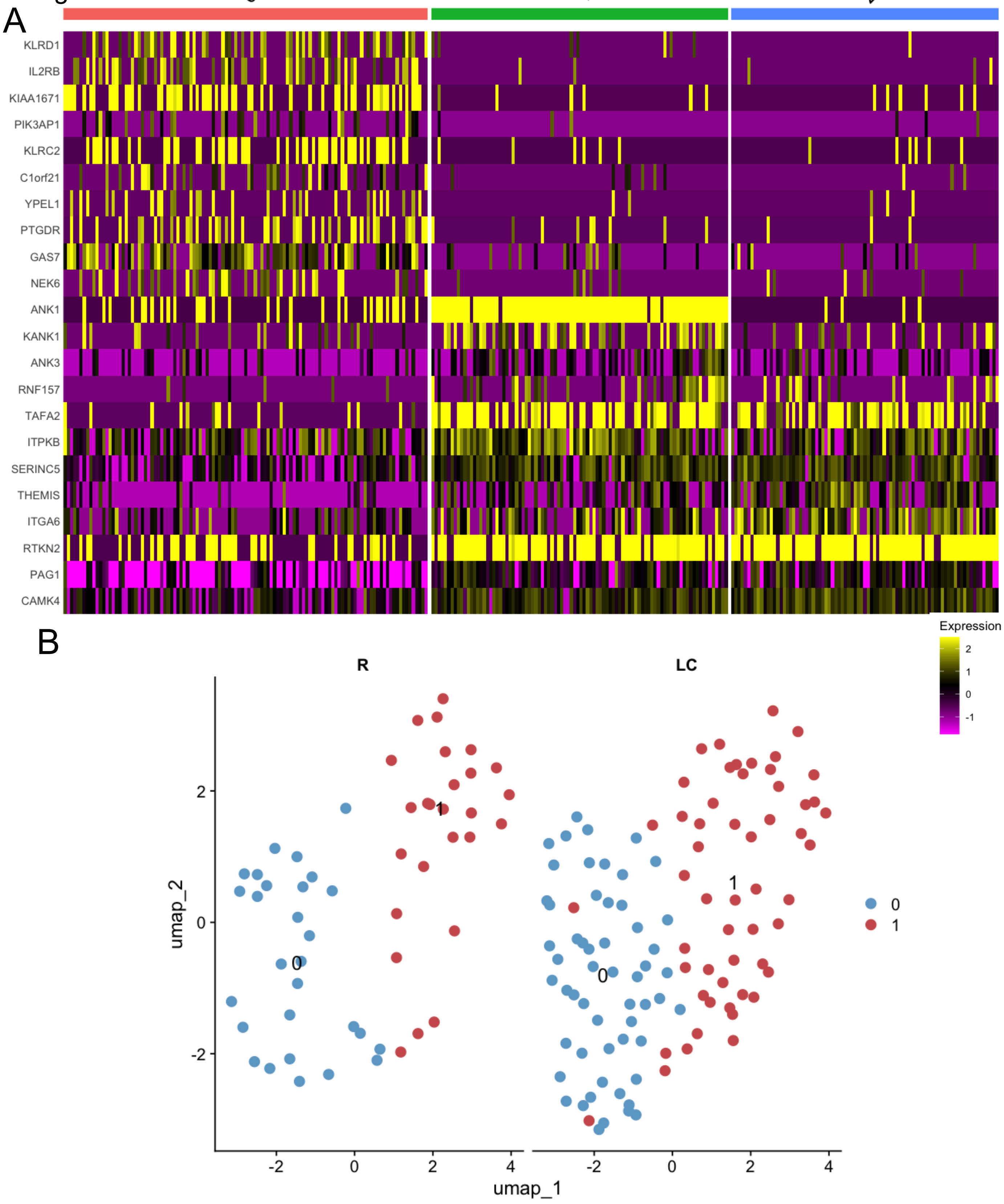

Fig S6. (**A**) Heatmap illustrating gene expression profiles distinguishing three subsets of gamma delta T cells. plot illustrating the expression of the genes associated with hormone-related receptor genes across different PBMC clusters. (**B**) UMAP projection of different subsets of platelets.

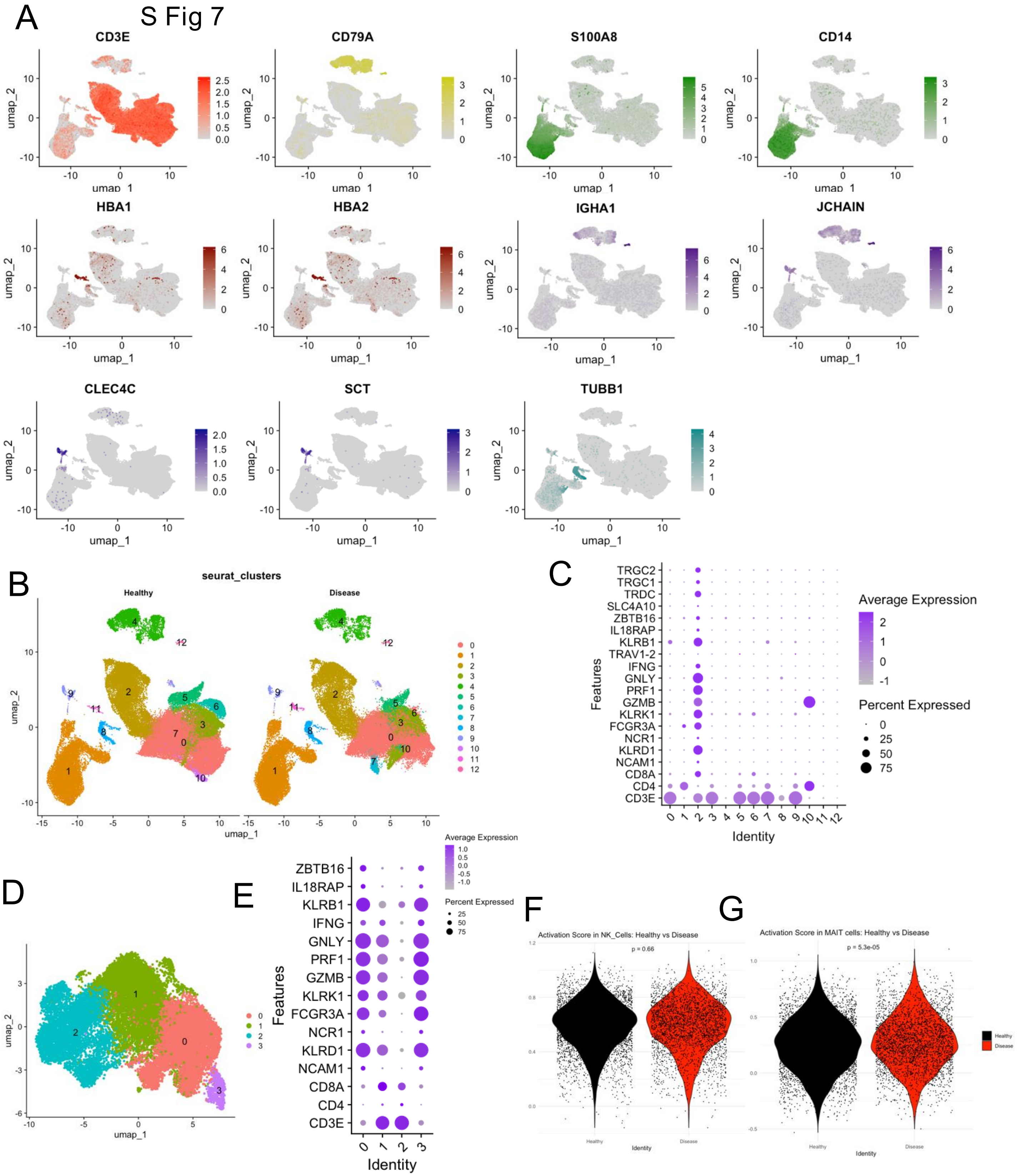

Fig S7. (**A**) Feature plots showing the expression of canonical genes associated with different immune cell clusters. (**B**) UMAP projection of merged PBMCs from healthy individuals and ME/CFS patients (Disease). (**C**) Bubble plot illustrating the expression of genes associated with MAIT cells, γδ T cells and NK cells across different immune clusters. (**D**) UMAP projection of cellular subsets within cluster 2, which contains both NK and MAIT cells. (**E**) Bubble plot showing the expression of MAIT- and NK cell–associated genes across subclusters within cluster 2. (**F**) Distribution of NK cell activation and (**G**) MAIT cell module scores in healthy individuals and patients with ME/CFS.

S Fig 8

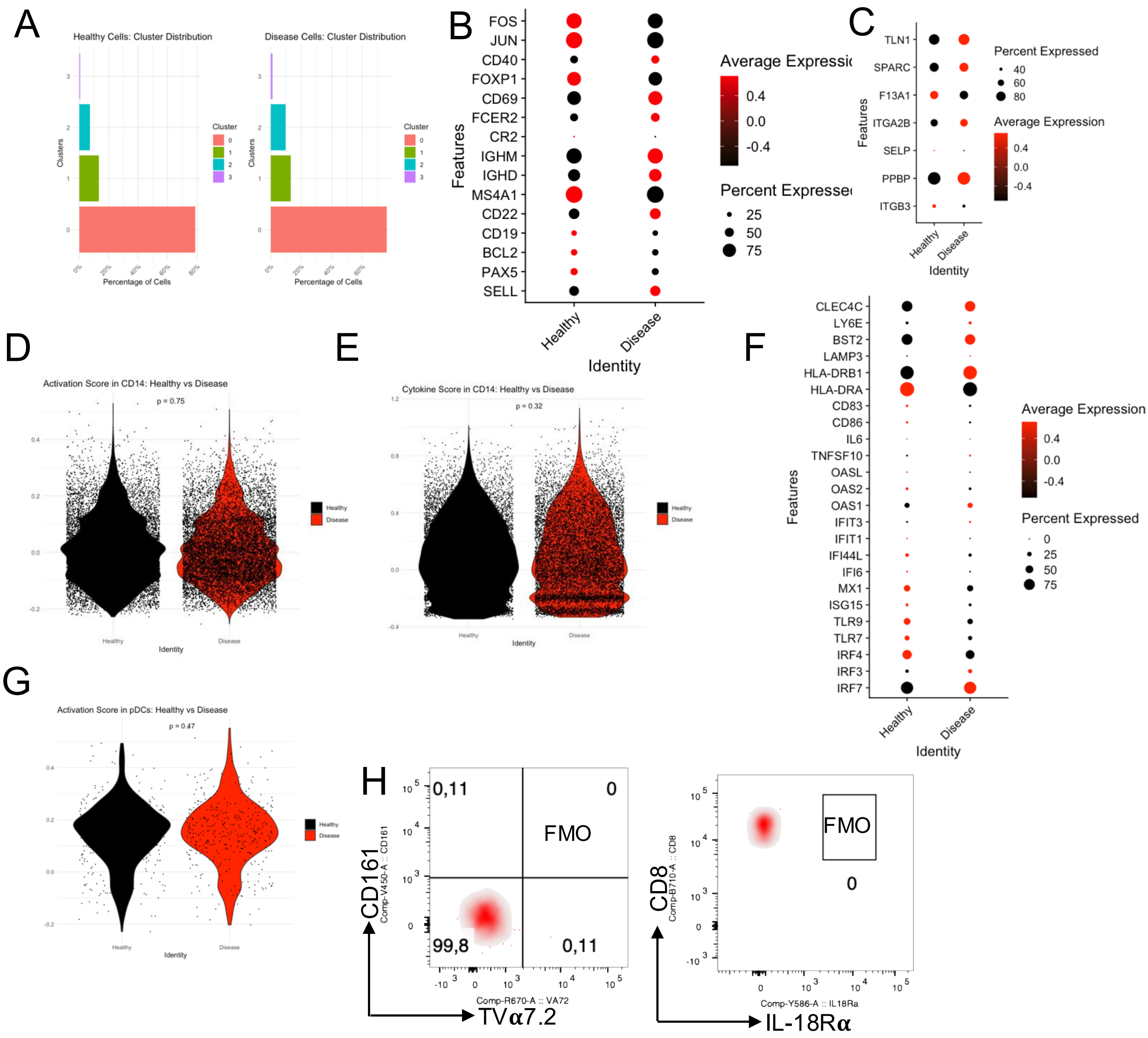

Fig S8. (**A**) Bar plots showing the percentage distribution of cluster 2 subclusters in healthy individuals and ME/CFS patients. (**B**) Bubble plots displaying differentially expressed genes associated with B cell and (**C**) platelet function in healthy individuals and ME/CFS patients. Distribution of (**D**) monocyte phagocytic activity and (**E**) cytokine module scores in healthy individuals and patients with ME/CFS. (**F**) Bubble plot depicting differentially expressed genes associated with pDCs function in healthy individuals and patients with ME/CFS. (**G**) Distribution of pDCs activation module scores comparing healthy individuals and ME/CFS patients. (**H**) Flow cytometry plots of fluorescence minus one (FMO) for CD161,TV**α**7.2, and IL-18R**α**.
